# Prenatal and Early Postnatal Odorant Exposure Heightens Odor-Evoked Mitral Cell Responses in the Mouse Olfactory Bulb

**DOI:** 10.1523/ENEURO.0129-17.2017

**Published:** 2017-09-26

**Authors:** Annie Liu, Nathaniel N. Urban

**Affiliations:** 1 Center for Neuroscience at the University of Pittsburgh, University of Pittsburgh, Pittsburgh, PA; 2 Center for the Neural Basis of Cognition, Pittsburgh, PA; 3 Department of Biological Sciences, Carnegie Mellon University, Pittsburgh, PA; 4 Department of Neurobiology, University of Pittsburgh, Pittsburgh, PA

**Keywords:** mitral cells, olfaction, olfactory bulb, plasticity

## Abstract

Early sensory experience shapes the anatomy and function of sensory circuits. In the mouse olfactory bulb (OB), prenatal and early postnatal odorant exposure through odorized food (food/odorant pairing) not only increases the volume of activated glomeruli but also increases the number of mitral and tufted cells (M/TCs) connected to activated glomeruli. Given the importance of M/TCs in OB output and in mediating lateral inhibitory networks, increasing the number of M/TCs connected to a single glomerulus may significantly change odorant representation by increasing the total output of that glomerulus and/or by increasing the strength of lateral inhibition mediated by cells connected to the affected glomerulus. Here, we seek to understand the functional impact of this long-term odorant exposure paradigm on the population activity of mitral cells (MCs). We use viral expression of GCaMP6s to examine odor-evoked responses of MCs following prenatal and early postnatal odorant exposure to two dissimilar odorants, methyl salicylate (MS) and hexanal, which are both strong activators of glomeruli on the dorsal OB surface. Previous work suggests that odor familiarity may decrease odor-evoked MC response in rodents. However, we find that early food-based odorant exposure significantly changes MC responses in an unexpected way, resulting in broad increases in the amplitude, number, and reliability of excitatory MC responses across the dorsal OB.

## Significance Statement

The structure and output of the olfactory bulb (OB) circuit can be modified by odor experience throughout both development and adulthood. The highly specific organization of this system lends itself to detailed analyses of how experience can shape the architecture of sensory system responses. Previous work demonstrated that prenatal and early postnatal odorant exposure using a food-based paradigm increased the number of primary projection neurons connected to activated glomeruli. This increase may have significant effects on odorant representation and OB output. In this study, we focus on understanding how this odorant exposure paradigm impacts the odor-evoked responses of one population of primary OB output neurons, the mitral cells (MCs).

## Introduction

The olfactory bulb (OB) has a stereotyped structure, the organization of which is dictated in part by odorant receptor (OR) identity (Ressler et al., 1994; Vassar et al., 1994; Potter et al., 2001; Bozza et al., 2002; Treloar et al., 2002; Feinstein and Mombaerts, 2004; Komiyama and Luo, 2006). Olfactory sensory neurons (OSNs) send axons to the OB, where axons from OSNs expressing the same OR converge into roughly spherical structures called glomeruli. Each glomerulus contains dendrites of a cohort of juxtaglomerular and primary projection neurons, the mitral and tufted cells (M/TCs), together, these make up a glomerular module, the basic odor-coding unit of the OB. Each glomerular module thus has a genetic identity based on OR expression as well as a cohort of activating odorant ligands for the corresponding OR.

This OR identity-based organization facilitates the investigation of how specific olfactory experiences change the OB. Anatomic studies demonstrate that the OB circuitry is highly plastic and subject to experience-dependent structural changes throughout both development and adulthood. Odor exposure using a number of different conditioning paradigms increases glomerular volume (Woo et al., 1987; Todrank et al., 2011; Dias and Ressler, 2014; Morrison et al., 2015). Combined prenatal and early postnatal odor exposure increases both glomerular volume and associated M/TC number (Todrank et al., 2011; Liu et al., 2016), while early aversive conditioning accelerates the rate of glomerular convergence (Kerr and Belluscio, 2006). Early postnatal passive odorant exposure has also been shown to decrease cell turnover in the glomerular and granule cell layers (Woo et al., 2006). In adult mice, aversive odorant conditioning increases OSN number and glomerular volume, these changes are reversed following the extinguishing of the learned aversive response (Morrison et al., 2015). These studies use different conditioning or exposure paradigms, yet they all have significant effects of OB circuitry, suggesting that several distinct mechanisms may influence the development and maintenance of OB structure. These large modifications to OB circuitry may in turn have significant effects on the representation and processing of odorants in the OB.

Odor-evoked activity in the rodent OB is also influenced by odorant experience and exposure. Rodents demonstrate both acute and chronic reductions in the amplitude and probability of odor-evoked excitatory M/TC responses following repeated odor presentation (Chaudhury et al., 2010; Kato et al., 2012). In rats, early postnatal odorant exposure paired with positive somatosensory stimuli decreases the number of excitatory M/TC responses elicited by the learned odorant while leaving responses to unassociated odorants unchanged (Wilson et al., 1985, 1987). However, the relationship between experience-dependent anatomic circuit changes and odor-evoked activity in the OB is not well understood, as there is significant interstudy variation in the delivery, context, and timing of odorant exposure or conditioning.

Here, we use an odor exposure paradigm with a previously characterized anatomic correlate to understand how odor representation may be affected by these changes in circuit structure. Todrank et al. (2011) and Liu et al. (2016) found that food-based prenatal and early postnatal odorant exposure increases glomerulus volume and the number of M/TCs corresponding to activated glomerular modules, while increasing behavioral preference for the odor that was paired with food. Given that a single odorant activates multiple glomeruli across the OB, these observed changes in glomerular volume and M/TC number could be generalized to many glomeruli and thus have a large distributed impact on odor representation in the OB. We investigate how early chronic food-based odorant exposure, a paradigm known to change OB anatomy, impacts the odor-evoked responses of MCs in the dorsal mouse OB. Surprisingly, we find that odor-evoked calcium transients in MCs are broadly enhanced rather than reduced in this food pairing paradigm.

## Materials and Methods

All animal procedures were performed in accordance with the University of Pittsburgh animal care committee's regulations.

### Animals and surgical methods

All experiments were done in male and female M72-IRES-tauGFP mice. Imaging was done on adult mice, at five to eight weeks of age. During all surgical procedures, animals were anesthetized using a ketamine/xylazine mixture, provided analgesia using carprofen injections, and maintained at 37°C body temperature. Expression of GCaMP6s was achieved through injection of 1 µl of AAV-hsyn-GCaMP6s ∼250 µm underneath the pial surface in the dorsal posterior OB surface. Animals were allowed to recover for two weeks, after which they were used for acute *in vivo* anesthetized imaging experiments. For acute imaging, a 2-mm diameter craniotomy was made over the dorsal posterior OB and covered with low melting point agarose. A coverslip was secured over the craniotomy using dental cement to minimize z-plane movement. Imaging was done in anesthetized animals maintained at 37°C body temperature.

### Odorant exposure

Prenatal and early postnatal odorant exposure was performed on M72-IRES-tauGFP mice as detailed in Todrank et al. (2011) and Liu et al. (2016). Two qualitatively distinct odorants were used for odor-exposure groups. Methyl salicylate (MS) has a wintergreen scent and activates both olfactory and trigeminal responses, while hexanal has a green grass scent and activates only olfactory receptors. Both odorants are strong activators of glomeruli on the dorsal OB surface in rats and mice, with different but overlapping activation areas (Glomerular Activity Response Archive, Michael Leon, gara.bio.uci.edu/index.jsp; Wachowiak and Cohen, 2001, 2003). We exposed animals to these odors by adding these odors to the food provided to these animals, as described previously (Todrank et al., 2011; Liu et al., 2016), a manipulation that results in altered preference for the conditioned food and altered glomerular module structure.

**Figure 1.**
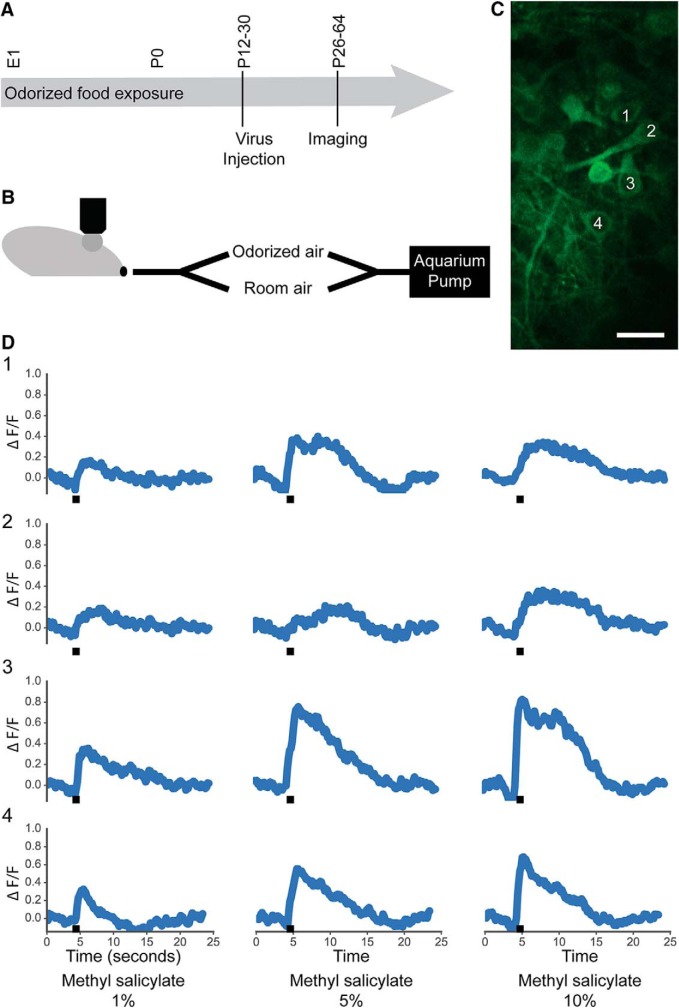
Odor-evoked calcium responses in the MC layer. ***A***, Odorized food exposure lasted through the entirety of gestation and the postnatal period until imaging. Virus injection into the dorsal OB was done at P12-P30. Imaging was performed two to three weeks after virus injection. ***B***, The dorsal OB was imaged during stimulus presentation using a custom-built 2-channel olfactometer with airflow provided by an aquarium pump. ***C***, The MC layer was imaged with manual ROI selection. Four cells are labeled, with corresponding odor-evoked responses shown in ***D***. White scale bar: 25 µm. Black bar indicates 1-s odor stimulus.

Food was mixed with either MS (mint) or hexanal (1% by volume) and dried under a fume hood for 3 d. Breeding pairs were fed with either control, mint-scented, or hexanal-scented food for the duration of gestation and nursing (Fig. 1*A*). Litters were subsequently weaned onto and continuously fed with either control, mint-scented, or hexanal-scented food until acute calcium imaging experiments. Both male and female mice were used for imaging. Breeding pairs were weighed regularly to ensure that odorized food did not interfere with food consumption. Odorant-exposed litters were not of substantially different weights from control litters.

### Stimulus delivery

Each trial consisted of 4-s room air, 1-s odorized air, and 25-s room air. Each odorant was presented four times in pseudorandom stimulus order and with intertrial interval length of at least 1 min. Stimuli consisted of 14 odorants: eight odorants diluted in mineral oil at different concentrations [1%: isoamyl acetate (IAA), hexanal, MS, ethyl butyrate (EB), propionic acid (PA), hexanone, acetophenone (AP), and 2-OH acetophenone (THA); 5%: IAA, hexanal, MS; 10%: IAA, hexanal, MS]. All odorants are known dorsal OB ligands. Odorants were delivered using a custom built olfactometer controlled via TTL input from a HEKA ITC-18 external DA/AD/TTL device run by IGOR Pro (RRID:SCR_000325; Fig. 1*B*).

### Imaging

Two-photon imaging was done in the MC layer ∼150-225 μm under the pial surface using a VIVO 2-Photon system from 3I Intelligent Imaging Innovations and SlideBook Imaging software. Image capture rate was 6-9 Hz.

### Analysis

The dataset consists of 863 putative mitral cells (MCs) from 17 mice (225 cells from one female and four male control mice, 269 cells from two female and four male mint-exposed mice, and 369 cells from two female and four male hexanal exposed) for a total of 12,082 cell-odor pairs. To select regions of interest (ROIs), cell somata in each field of view were traced manually using SlideBook software and avoiding intersecting cell processes as determined by Z-stacks of each field of view (Fig. 1*C*). Presence or lack of odor-evoked response was not taken into account when identifying cells. Rather, all identified fluorescent cells were included. Raw intensity values were calculated using SlideBook software. ΔF/F traces were calculated using the average baseline intensity from the 20 frames before stimulus onset at 4 s after start of the trial. Traces were detrended by subtracting a polynomial fit from the calculated ΔF/F trace (Fig. 1*D*). We fit the 20 frames before odor onset (∼3 s before odor onset) and the last 45 frames (∼19 s after odor end) of each trace to a second degree polynomial and subtracted this fit from each trace. Response threshold was set at 3 SD away from baseline. Responses were only counted as successful if above this threshold, otherwise response was recorded as 0. All analysis and visualization was done using Python (RRID:SCR_008394). Some statistical tests were performed using GraphPad Prism (RRID:SCR_002798).

## Results

### Majority of MC odor-evoked responses were excitatory

**Figure 2.**
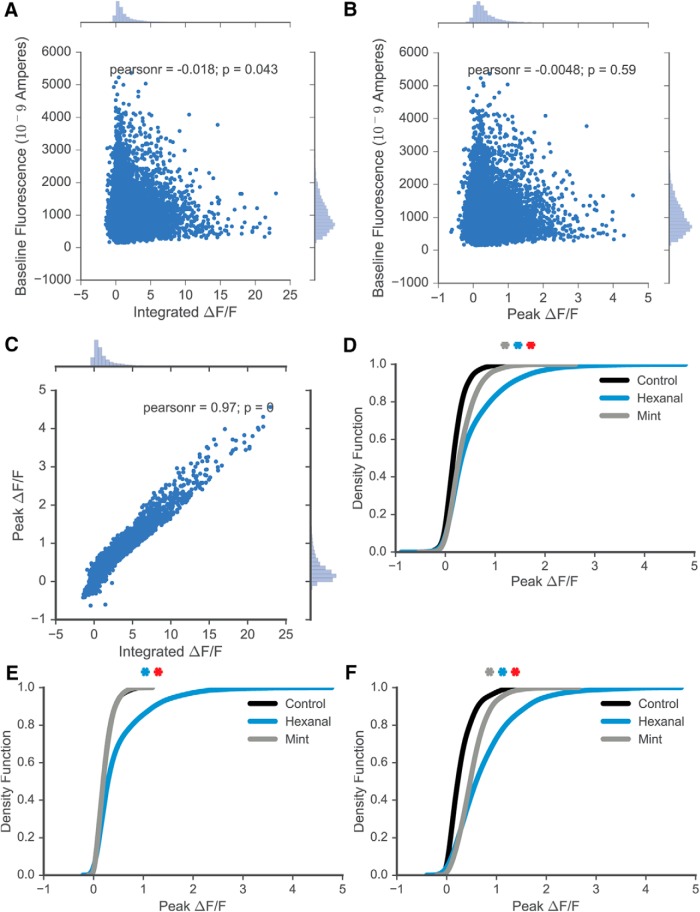
Response characteristics. ***A***, Integrated ΔF/F and baseline fluorescence are not correlated. ***B***, Peak ΔF/F and baseline fluorescence are not correlated. ***C***, Peak and integrated ΔF/F are strongly correlated. ***D***, Kernel density estimation (KDE) describing response distribution across all cells and odorants in control, hexanal-exposed, and mint-exposed mice (statistically significant distributions between groups). ***E***, KDE describing response distribution across cells in control, hexanal-exposed, and mint-exposed male mice. ***F***, KDE describing response distribution across cells in control, hexanal-exposed, and mint-exposed female mice. Gray asterisk, statistically significant difference between mint and control groups; blue asterisk, statistically significant difference between hexanal and control; red asterisk, statistically significant difference between hexanal and mint.

MC odor-evoked responses were dominated by excitatory responses, constituting 11,089 cell-odor pairs out of 12,082 total cell-odor pairs or ∼92% of all measured responses. Both peak ΔF/F (peak amplitude of ΔF/F response) and integral of odor-evoked response (integrated ΔF/F) were calculated. A strong correlation between the peak and integral of individual responses was observed (Pearson’s *r* = 0.97; Fig. 2*C*), but there was no significant correlation between initial baseline intensity and either the peak (Pearson’s *r* = -0.0048) or integral of responses (Pearson’s *r* = -0.018; Fig. 2*A*,*B*). Thus, all data were pooled regardless of initial baseline intensity. Given the strong correlation between the peak and integral of odor-evoked response, subsequent data regarding odorant-evoked responses are shown as peak values. The distribution of all responses to all odorants are visualized in Figure 2*D*, with significant statistical differences observed between the response density functions of MCs from control, hexanal-exposed, and mint-exposed animals (Kruskal--Wallis test with Tukey’s multiple comparisons test; *p* = 6.65E-199; adjusted *p* values between groups approximated 0).

We observe significant cell population differences in response between the control and each of the odor-exposed groups. We next assessed potential differences in odor-evoked response based on subject sex. When examining only male mice (Fig. 2*E*), we find that the response distributions are different between hexanal-exposed and control mice (Kruskal--Wallis, *p* = 1.13E-66), but not different between mint-exposed and control mice (Kruskal--Wallis, *p* = 0.69). However, when examining cell responses from female mice (Fig. 2*F*), we find that the distributions of cell responses from both groups of odor-exposed animals significantly differ from the distribution of control mouse responses (Kruskal--Wallis; control vs hexanal exposed: *p* = 1.29E-97; control vs mint exposed: *p* = 1.02E-81). The sample size of female mice (*n* = 1 control, two mint-exposed and two hexanal-exposed female mice) was not sufficient to draw specific conclusions about the role of sex in amplifying odor-evoked responses following early odorant exposure. Given the small animal number, we cannot determine if these intergroup differences are due to interanimal variability or sexual dimorphism. Male and female mice pooled across groups did not have significantly different ages (Student’s *t* test; male vs female; *n* = 12 vs 5; mean P48.67 ± 6.56 vs P41.8 ± 9.24; *p* = 0.57).

### Amplitude of excitatory response increases following early odorant exposure

**Figure 3.**
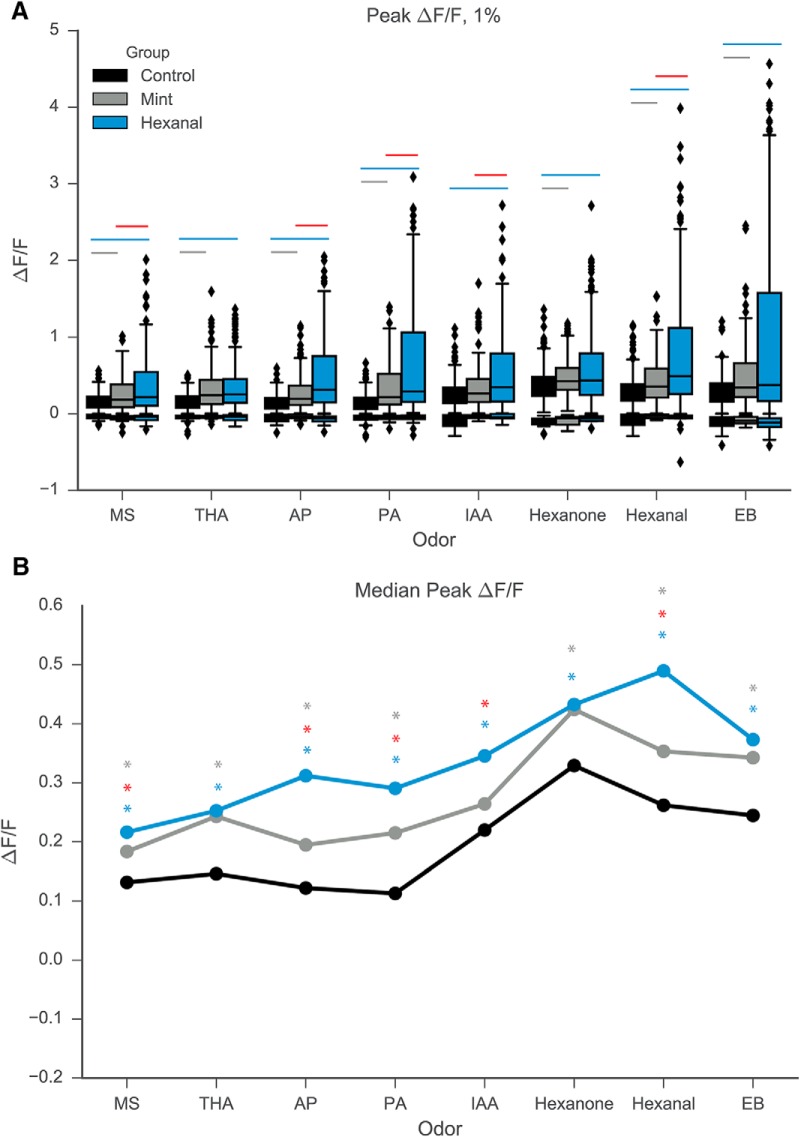
Odor exposure increases median Peak ΔF/F of MC response to odorants at 1% concentration by volume. ***A***, Boxplot describing distribution of peak odor-evoked ΔF/F of MC response to odorants at 1% concentration by volume. ***B***, Median peak ΔF/F across all odorants at 1% concentration by volume. Gray asterisk, statistically significant difference between mint and control groups; blue asterisk, statistically significant difference between hexanal and control; red asterisk, statistically significant difference between hexanal and mint.

**Figure 4.**
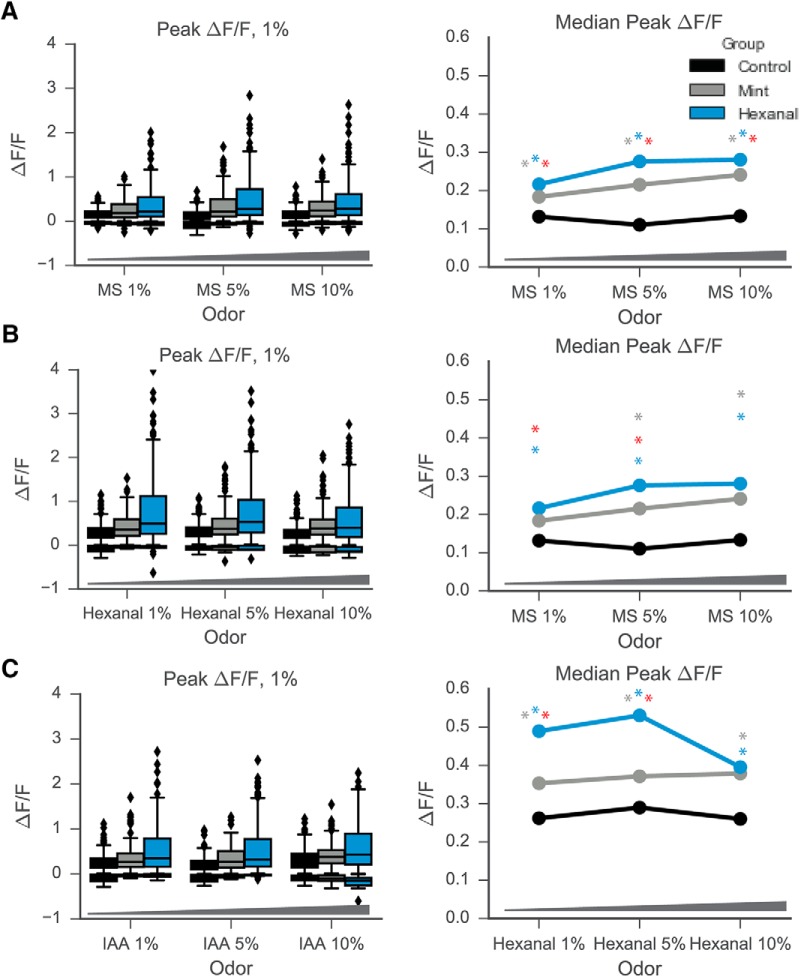
Odor exposure increases median Peak ΔF/F of MC response to odorants across concentrations. Mint-exposed and hexanal-exposed MCs have higher medians of peak ΔF/F across all concentrations of MS (***A***), IAA (***B***), and hexanal (***C***). There were no significant differences in response distribution between concentrations for any exposure group.

Several additional features of the odor-evoked response change significantly following early odorant exposure, namely amplitude, number, and reliability of response. The amplitude of excitatory odor-evoked responses increased significantly for cells in both odor-exposed groups as compared to cells in the control animals (Fig. 3*A*). Figure 3*B* shows clear differences in median ΔF/F across all odorants at 1% concentration for control versus hexanal- and mint-exposed and for hexanal versus mint-exposed. This increase in response amplitude is not odor-specific, but rather is observed for almost all odors and concentrations in the odor stimulus panel (Figs. 3, 4). Descriptive and odor-specific group comparison statistics can be found in Table 1.

**Table 1.** Comparisons of excitatory response amplitude, 1% odorant concentration

Comparison	Test	*p* value	Significant	Median control (C)	Median mint (M)	Median hexanal (H)
**Peak ΔF/F amplitude**				****C;******N******= 225****	****M;******N******= 268****	****H;******N******= 369****
**IAA 10%** **all groups**	**Kruskal--Wallis**	**<0.0001**	**Yes**	**0.216**	**0.3547**	**0.3697**
**IAA 10%; C vs M**	**Dunn's**	**<0.0001**	**Yes**	**0.216**	**0.3547**	
**IAA 10%; C vs H**	**Dunn's**	**<0.0001**	**Yes**	**0.216**		**0.3697**
IAA 10%; H vs M	Dunn's	0.0967	No		0.3547	0.3697
**IAA 1%; all groups**	**Kruskal--Wallis**	**<0.0001**	**Yes**	**0.2018**	**0.2389**	**0.3306**
**IAA 1%; C vs M**	**Dunn's**	**0.0745**	**No**	**0.2018**	**0.2389**	
**IAA 1%; C vs H**	**Dunn's**	**<0.0001**	**Yes**	**0.2018**		**0.3306**
**IAA 1%; H vs M**	**Dunn's**	**0.0001**	**Yes**		**0.2389**	**0.3306**
**AP all groups**	**Kruskal--Wallis**	**<0.0001**	**Yes**	**0.1081**	**0.1837**	**0.2822**
**AP; C vs M**	**Dunn's**	**<0.0001**	**Yes**	**0.1081**	**0.1837**	
**AP; C vs H**	**Dunn's**	**<0.0001**	**Yes**	**0.1081**		**0.2822**
**AP; H vs M**	**Dunn's**	**<0.0001**	**Yes**		**0.1837**	**0.2822**
**MS 10%** **all groups**	**Kruskal--Wallis**	**<0.0001**	**Yes**	**0.1026**	**0.1961**	**0.2566**
**MS 10%; C vs M**	**Dunn's**	**<0.0001**	**Yes**	**0.1026**	**0.1961**	
**MS 10%; C vs H**	**Dunn's**	**<0.0001**	**Yes**	**0.1026**		**0.2566**
**MS 10%; H vs M**	**Dunn's**	**0.0143**	**Yes**		**0.1961**	**0.2566**
**IAA 5%; all groups**	**Kruskal--Wallis**	**<0.0001**	**Yes**	**0.1404**	**0.2386**	**0.2973**
**IAA 5%; C vs M**	**Dunn's**	**<0.0001**	**Yes**	**0.1404**	**0.2386**	
**IAA 5%; C vs H**	**Dunn's**	**<0.0001**	**Yes**	**0.1404**		**0.2973**
**IAA 5%; H vs M**	**Dunn's**	**0.0023**	**Yes**		**0.2386**	**0.2973**
**Hexanal 1%; all groups**	**Kruskal--Wallis**	**<0.0001**	**Yes**	**0.2409**	**0.3421**	**0.4718**
**Hexanal 1%; C vs M**	**Dunn's**	**<0.0001**	**Yes**	**0.2409**	**0.3421**	
**Hexanal 1%; C vs H**	**Dunn's**	**<0.0001**	**Yes**	**0.2409**		**0.4718**
**Hexanal 1%; H vs M**	**Dunn's**	**0.0001**	**Yes**		**0.3421**	**0.4718**
**EB all groups**	**Kruskal--Wallis**	**<0.0001**	**Yes**	**0.2262**	**0.3254**	**0.3204**
**EB; C vs M**	**Dunn's**	**<0.0001**	**Yes**	**0.2262**	**0.3254**	
**EB; C vs H**	**Dunn's**	**<0.0001**	**Yes**	**0.2262**		**0.3204**
EB; H vs M	Dunn's	0.6847	No		0.3254	0.3204
**MS 1%; all groups**	**Kruskal--Wallis**	**<0.0001**	**Yes**	**0.1167**	**0.1641**	**0.1933**
**MS 1%; C vs M**	**Dunn's**	**0.0028**	**Yes**	**0.1167**	**0.1641**	
**MS 1%; C vs H**	**Dunn's**	**<0.0001**	**Yes**	**0.1167**		**0.1933**
**MS 1%; H vs M**	**Dunn's**	**0.0026**	**Yes**		**0.1641**	**0.1933**
**PA all groups**	**Kruskal--Wallis**	**<0.0001**	**Yes**	**0.0733**	**0.2004**	**0.2511**
**PA; C vs M**	**Dunn's**	**<0.0001**	**Yes**	**0.0733**	**0.2004**	
**PA; C vs H**	**Dunn's**	**<0.0001**	**Yes**	**0.0733**		**0.2511**
**PA; H vs M**	**Dunn's**	**0.0244**	**Yes**		**0.2004**	**0.2511**
**MS 5%; all groups**	**Kruskal--Wallis**	**<0.0001**	**Yes**	**0.0777**	**0.1796**	**0.2503**
**MS 5%; C vs M**	**Dunn's**	**<0.0001**	**Yes**	**0.0777**	**0.1796**	
**MS 5%; C vs H**	**Dunn's**	**<0.0001**	**Yes**	**0.0777**		**0.2503**
**MS 5%; H vs M**	**Dunn's**	**<0.0001**	**Yes**		**0.1796**	**0.2503**
**Hexanone; all groups**	**Kruskal--Wallis**	**<0.0001**	**Yes**	**0.3185**	**0.4161**	**0.421**
**Hexanone; C vs M**	**Dunn's**	**<0.0001**	**Yes**	**0.3185**	**0.4161**	
**Hexanone; C vs H**	**Dunn's**	**<0.0001**	**Yes**	**0.3185**		**0.421**
Hexanone; H vs M	Dunn's	0.8052	No		0.4161	0.421
**Hexanal 10%; all groups**	**Kruskal--Wallis**	**<0.0001**	**Yes**	**0.2438**	**0.3768**	**0.3778**
**Hexanal 10%; C vs M**	**Dunn's**	**<0.0001**	**Yes**	**0.2438**	**0.3768**	
**Hexanal 10%; C vs H**	**Dunn's**	**<0.0001**	**Yes**	**0.2438**		**0.3778**
Hexanal 10%; H vs M	Dunn's	0.9975	No		0.3768	0.3778
**THA all groups**	**Kruskal--Wallis**	**<0.0001**	**Yes**	**0.1171**	**0.2183**	**0.2415**
**THA; C vs M**	**Dunn's**	**<0.0001**	**Yes**	**0.1171**	**0.2183**	
**THA; C vs H**	**Dunn's**	**<0.0001**	**Yes**	**0.1171**		**0.2415**
ThA; H vs M	Dunn's	0.2795	No		0.2183	0.2415
**Hexanal 5%; all groups**	**Kruskal--Wallis**	**<0.0001**	**Yes**	**0.2758**	**0.3534**	**0.5212**
**Hexanal 5%; C vs M**	**Dunn's**	**0.0001**	**Yes**	**0.2758**	**0.3534**	
**Hexanal 5%; C vs H**	**Dunn's**	**<0.0001**	**Yes**	**0.2758**		**0.5212**
**Hexanal 5%; H vs M**	**Dunn's**	**<0.0001**	**Yes**		**0.3534**	**0.5212**

Statistical test results of excitatory response amplitudes of cells from control, mint-, and hexanal-exposed groups in response to odorants at 1% concentration. Data shown in Figure 3.

Within-group differences in responses to the same odorant were also observed across concentrations (Fig. 4). However, these concentration differences were largely similar between the control, mint-exposed, and hexanal-exposed groups, suggesting that the increased responsiveness seen following odor exposure was not specific to a single concentration. There were two combinations of odorants and concentrations for which significant differences were observed in all three exposure groups, two additional combinations with significant differences observed in only the control group, 1 additional observed in the mint-exposed group, and two additional observed in the hexanal-exposed group (Kruskal--Wallis test with Tukey’s multiple comparisons). Full details about statistical comparisons available in Table 2.

**Table 2.** Comparisons of excitatory response amplitude, multiple concentrations

Comparison	Test	*p* value	Significant	Median control (C)	Median mint (M)	Median hexanal (H)
**Peak ΔF/F amplitude**				****C;******N******= 225****	****M;******N******= 268****	****H;******N******= 369****
**Control group**						
**MS all conc.**	**Kruskal--Wallis**	**0.0586**	**No**	**0.1167**	**0.0777**	**0.1026**
**MS; 1% vs 5%**	**Dunn's**	**0.0508**	**Yes**	**0.1167**	**0.0777**	
**MS; 1% vs 10%**	**Dunn's**	**0.4478**	**No**	**0.1167**		**0.1026**
**MS; 5% vs 10%**	**Dunn's**	**0.4695**	**No**		**0.0777**	**0.1026**
**IAA all conc.**	**Kruskal--Wallis**	**<0.0001**	**Yes**	**0.2018**	**0.1404**	**0.216**
**IAA; 1% vs 5%**	**Dunn's**	**0.0028**	**Yes**	**0.2018**	**0.1404**	
**IAA; 1% vs 10%**	**Dunn's**	**0.768**	**No**	**0.2018**		**0.216**
**IAA; 5% vs 10%**	**Dunn's**	**0.0001**	**Yes**		**0.1404**	**0.216**
**Hexanal; all conc.**	**Kruskal--Wallis**	**0.0214**	**Yes**	**0.2409**	**0.2758**	**0.2438**
**Hexanal; 1% vs 5%**	**Dunn's**	**0.1657**	**No**	**0.2409**	**0.2758**	
**Hexanal; 1% vs 10%**	**Dunn's**	**0.8025**	**No**	**0.2409**		**0.2438**
**Hexanal; 5% vs 10%**	**Dunn's**	**0.0206**	**Yes**		**0.2758**	**0.2438**
**Mint group**						
**MS; all conc.**	**Kruskal--Wallis**	**0.206**	**No**	**0.1642**	**0.1796**	**0.1961**
**MS; 1% vs 5%**	**Dunn's**	**0.0194**	**Yes**	**0.1642**	**0.1796**	
**MS; 1% vs 10%**	**Dunn's**	**0.1692**	**No**	**0.1642**		**0.1961**
**MS; 5% vs 10%**	**Dunn's**	**0.7856**	**No**		**0.1796**	**0.1961**
**IAA; all conc.**	**Kruskal--Wallis**	**<0.000A1**	**Yes**	**0.2389**	**0.2386**	**0.3547**
**IAA; 1% vs 5%**	**Dunn's**	**0.9991**	**No**	**0.2389**	**0.2386**	
**IAA; 1% vs 10%**	**Dunn's**	**0.0001**	**Yes**	**0.2389**		**0.3547**
**IAA; 5% vs 10%**	**Dunn's**	**0.0001**	**Yes**		**0.2386**	**0.3547**
**Hexanal; all conc.**	**Kruskal--Wallis**	**0.3426**	**No**	**0.3421**	**0.3534**	**0.3768**
**Hexanal; 1% vs 5%**	**Dunn's**	**0.7615**	**No**	**0.3421**	**0.3534**	
**Hexanal; 1% vs 10%**	**Dunn's**	**0.3775**	**No**	**0.3421**		**0.3768**
**Hexanal; 5% vs 10%**	**Dunn's**	**0.9178**	**No**		**0.3534**	**0.3768**
**Hexanal group**						
**MS; all conc.**	**Kruskal--Wallis**	**0.0184**	**Yes**	**0.1933**	**0.2503**	**0.2566**
**MS; 1% vs 5%**	**Dunn's**	**0.0146**	**Yes**	**0.1933**	**0.2503**	
**MS; 1% vs 10%**	**Dunn's**	**0.2718**	**No**	**0.1933**		**0.2566**
**MS; 5% vs 10%**	**Dunn's**	**0.5645**	**No**		**0.2503**	**0.2566**
**IAA; all conc.**	**Kruskal--Wallis**	**0.0433**	**Yes**	**0.3306**	**0.2973**	**0.3697**
**IAA; 1% vs 5%**	**Dunn's**	**0.8326**	**No**	**0.3306**	**0.2973**	
**IAA; 1% vs 10%**	**Dunn's**	**0.2488**	**No**	**0.3306**		**0.3697**
**IAA; 5% vs 10%**	**Dunn's**	**0.0425**	**Yes**		**0.2973**	**0.3697**
**Hexanal; all conc.**	**Kruskal--Wallis**	**<0.0001**	**Yes**	**0.4718**	**0.5212**	**0.3778**
**Hexanal; 1% vs 5%**	**Dunn's**	**0.3105**	**No**	**0.4718**	**0.5212**	
**Hexanal; 1% vs 10%**	**Dunn's**	**0.0137**	**Yes**	**0.4718**		**0.3778**
**Hexanal; 5% vs 10%**	**Dunn's**	**<0.0001**	**Yes**		**0.5212**	**0.3778**

Statistical test results of excitatory response amplitudes of cells from control, mint-, and hexanal-exposed groups in response to odorants at 1%, 5%, and 10% concentration. Data shown in Figure 4.

### Early odorant exposure increases the number and reliability of excitatory responses

**Figure 5.**
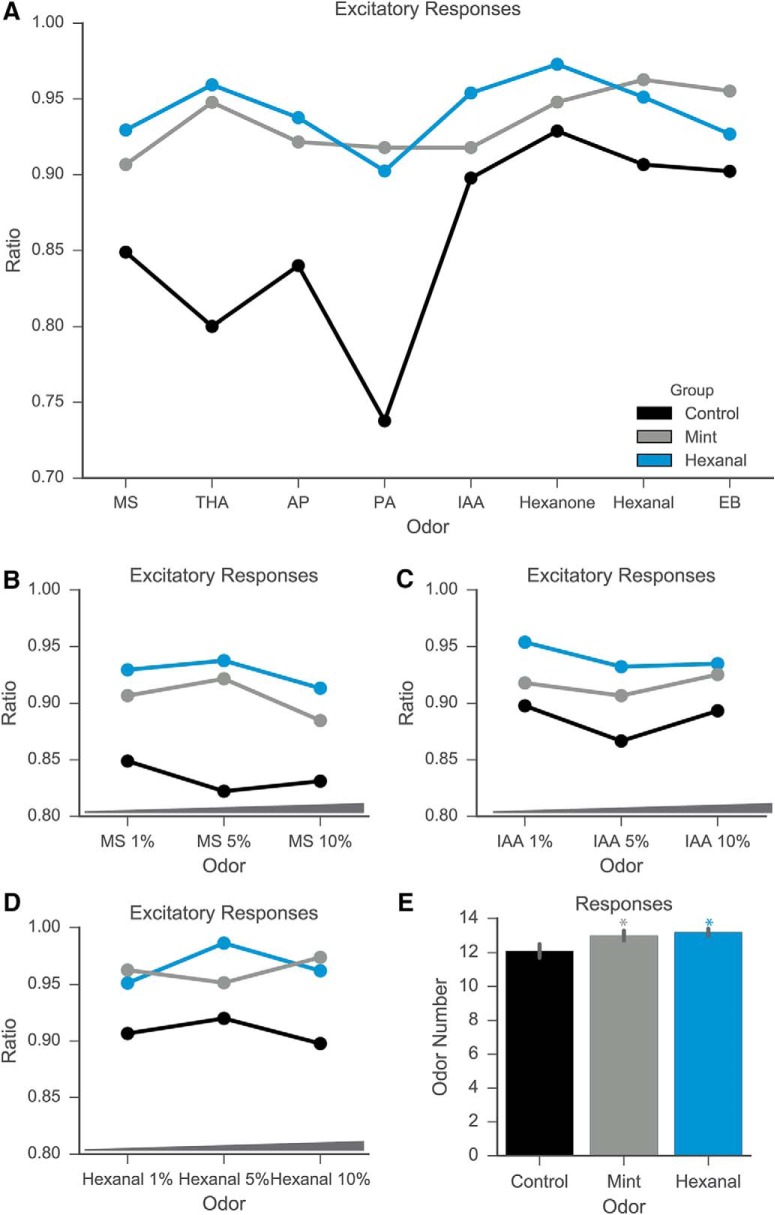
Odor exposure increases number of excitatory MC responses. ***A--D***, Ratio of above-threshold excitatory responses to all odor presentation trials across odorants. Odor-exposure groups had significantly higher ratio of excitatory responses as compared to control groups for odors at 1% concentration (***A***) and multiple concentrations (***B***--***D***). ***E***, MCs in all groups responded to a high number of odorants (median number of odorants: control, 13; mint exposed, 14; hexanal exposed, 14). Gray asterisk, statistically significant difference between mint and control groups; blue asterisk, statistically significant difference between hexanal and control groups.

The proportion of excitatory responses increases following prenatal and early postnatal odorant exposure (Fig. 5*A-D*; Table 3; ANOVA with Tukey’s multiple comparisons test; *p* < 0.0001; medians: control, 0.88; mint, 0.9235; hexanal, 0.9377; significant Tukey’s tests for control vs mint and control vs hexanal). The mint-exposed and hexanal-exposed groups both had a significantly larger proportion of excitatory responses than the control group. In addition, MCs of odor-exposed animals exhibited excitatory responses to more odorants than MCs of control animals (Fig. 5*E*; ANOVA with Tukey’s multiple comparisons test; *p* < 0.0001; medians: control, 13; mint, 14; hexanal, 14; significant Tukey’s tests for control vs mint and control vs hexanal). These measures indicate that odor exposure increases excitatory MC odor-evoked responses, both in number and in number of activating odors.

**Table 3.** Proportion of excitatory responses

Comparison	Test	*p* value	Significant	Median control (C)	Median mint (M)	Median hexanal (H)
**Ratio of excitatory responses**				****C;******N******= 14****	****M;******N******= 14****	****H;******N******= 14****
**All odors**	**ANOVA**	******p******< 0.0001****	**Yes**	**0.88**	**0.9235**	**0.9377**
**All odors; C vs M**	**Tukey's**	******p******< 0.0001****	**Yes**	**0.88**	**0.9235**	
**All odors; C vs H**	**Tukey's**	******p******< 0.0001****	**Yes**	**0.88**		**0.9377**
All odors; H vs M	Tukey's	*p* = 0.1313	No		0.9235	0.9377
**Ratio of activating** **odorants**				****C;******N******= 225****	****M;******N******= 268****	****H;******N******= 369****
**All groups**	**Kruskal--Wallis**	******p******< 0.0001****	**Yes**	**13**	**14**	**14**
**C vs M**	**Tukey's**	******p******< 0.0001****	**Yes**	**13**	**14**	
**C vs H**	**Tukey's**	******p******< 0.0001****	**Yes**	**13**		**14**
H vs M	Tukey's	p>0.9999	No		14	14

Comparisons of proportion of excitatory odor-evoked responses from control, mint-, and hexanal-exposed groups. Data shown in Figure 5.

**Figure 6.**
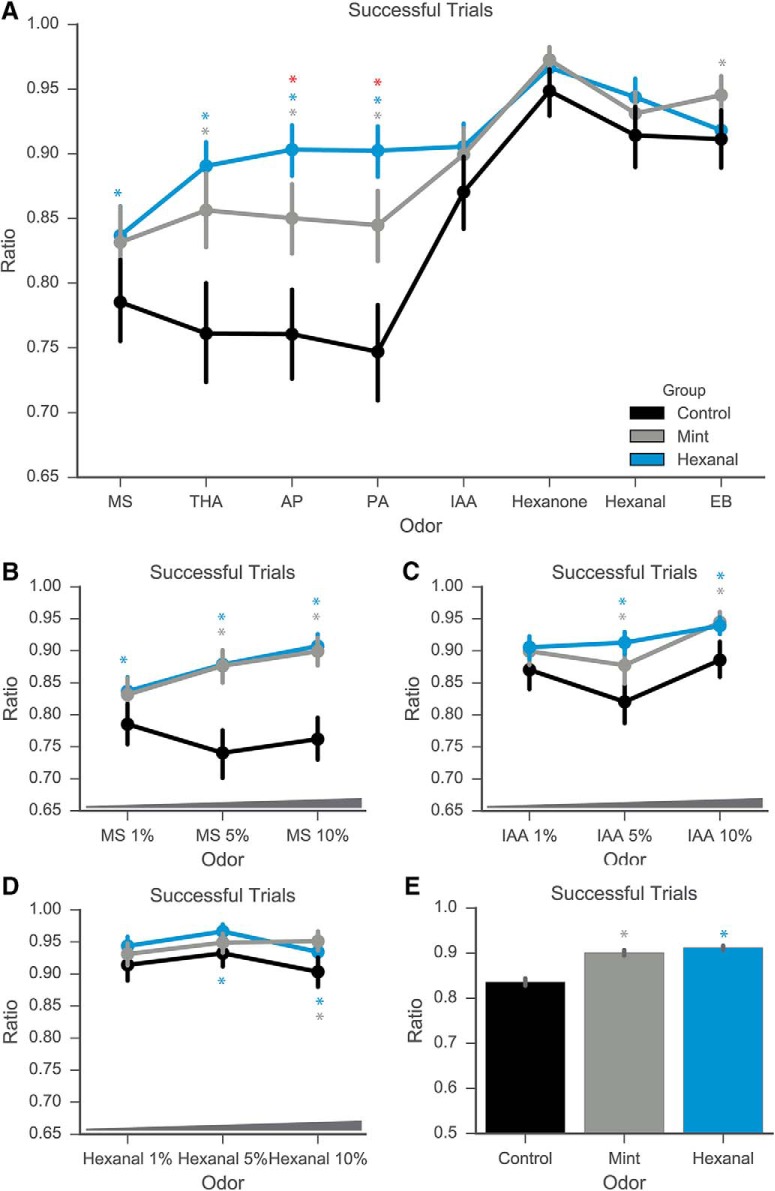
Odor exposure increases the rate of successful responses to odorant presentation. ***A--D***, Ratio of successful trials above threshold (3 SD above baseline) to total trials of odorant presentation increases in odor-exposed groups across specific odorants at 1% concentration (***A***) and multiple concentrations of MS (***B***) and IAA (***C***), but not hexanal (***D***). ***E***, Summed across all trials, odor-exposed groups had higher overall ratio of successful trials. Gray asterisk, statistically significant difference between mint and control groups; blue asterisk, statistically significant difference between hexanal and control; red asterisk, statistically significant difference between hexanal and mint.

Excitatory responses to certain odorants were also more reliable following odor-exposure, as measured by the proportion of successful trial presentations of each odorant (Fig. 6*A-D*; Table 4; data pooled across odorants; Kruskal--Wallis test with Dunn’s multiple comparisons test; *p* < 0.0001; medians: 1 for each group). As detailed in the Methods section, an excitatory response is defined as a peak ΔF/F at least 3 SD above average fluorescence intensity before stimulus onset. This increase in the reliability of MCs between control and odor-exposed groups (0.75 median control to 1 for each odor-exposed group) is significant for the following odorants: MS 1% (Kruskal--Wallis test; *p* = 0.0139), MS 5% (*p* < 0.0001), MS 10% (*p* < 0.0001), THA (*p* < 0.0001), AP (*p* < 0.0001), and PA (*p* < 0.0001; Fig. 6*A*; additional descriptive statistics available in Table 4). The odorants for which reliability increased following odorant exposure are the ones within the panel that elicited relatively weaker responses, as measured by comparisons of their median evoked peak ΔF/F (Figs. 3, 4). Median success values were not different between groups for other odorants, although comparisons of distributions differed – these values are described in Table 4. These data together show that early odorant exposure increases the proportion and reliability of excitatory responses in a manner not dependent on the identity of odorant used for exposure, much like the observed odor-nonspecific increases in excitatory response amplitude. Rather, the reliability of response increases in an odor-specific manner relative to the initial amplitude of odor-evoked response.

**Table 4.** Reliability of excitatory responses

Comparison	Test	*p* value	Median control (C)	Median mint (M)	Median hexanal (H)
**Ratio of successful trials**			****C;******N******= 225****	****M;******N******= 268****	****H;******N******= 369****
**All odors combined; all groups**	**Kruskal--Wallis**	**<0.0001**	**1**	**1**	**1**
**All odors combined; C vs M**	**Dunn's**	**<0.0001**	**1**	**1**	
**All odors combined; C vs H**	**Dunn's**	**<0.0001**	**1**		**1**
All odors combined; H vs M	Dunn's	0.0836		1	1
**AP all groups**	**Kruskal--Wallis**	**<0.0001**	**0.75**	**1**	**1**
**AP; C vs M**	**Dunn's**	**<0.0001**	**0.75**	**1**	
**AP; C vs H**	**Dunn's**	**<0.0001**	**0.75**		**1**
**AP; H vs M**	**Dunn's**	**0.0029**		**1**	**1**
**EB all groups**	**Kruskal--Wallis**	**0.0181**	**1**	**1**	**1**
**EB; C vs M**	**Dunn's**	**0.0151**	**1**	**1**	
EB; C vs H	Dunn's	0.5476	1		1
EB; H vs M	Dunn's	0.2351		1	1
Hexanal 1%; all groups	Kruskal--Wallis	0.0596	1	1	1
Hexanal 1%; C vs M	Dunn's	0.7821	1	1	
Hexanal 1%; C vs H	Dunn's	0.0562	1		1
Hexanal 1%; H vs M	Dunn's	0.6464		1	1
Hexanone; all groups	Kruskal--Wallis	0.1165	1	1	1
Hexanone; C vs M	Dunn's	0.1277	1	1	
Hexanone; C vs H	Dunn's	0.3668	1		1
Hexanone; H vs M	Dunn's	>0.9999		1	1
IAA 1% all groups	Kruskal--Wallis	0.0902	1	1	1
IAA 1%; C vs M	Dunn's	0.4431	1		1
IAA 1%; C vs H	Dunn's	0.0867	1	1	
IAA 1%; H vs M	Dunn's	>0.9999		1	1
**MS 1%** **all groups**	**Kruskal--Wallis**	**0.0139**	**0.75**	**1**	**1**
**MS 1%; C vs M**	**Dunn's**	**0.0621**	**0.75**	**1**	
**MS 1%; C vs H**	**Dunn's**	**0.0144**	**0.75**		**1**
MS 1%; H vs M	Dunn's	>0.9999		1	1
**PA all groups**	**Kruskal--Wallis**	**<0.0001**	**0.75**	**1**	**1**
**PA; C vs M**	**Dunn's**	**<0.0001**	**0.75**	**1**	
**PA; C vs H**	**Dunn's**	**<0.0001**	**0.75**		**1**
**PA; H vs M**	**Dunn's**	**0.0019**		**1**	**1**
**THA all groups**	**Kruskal--Wallis**	**<0.0001**	**0.75**	**1**	**1**
**THA; C vs M**	**Dunn's**	**<0.0001**	**0.75**	**1**	
**THA; C vs H**	**Dunn's**	**<0.0001**	**0.75**		**1**
THA; H vs M	Dunn's	0.9588		1	1
**MS 5%; all groups**	**Kruskal--Wallis**	**<0.0001**	**0.75**	**1**	**1**
**MS 5%; C vs M**	**Dunn's**	**<0.0001**	**0.75**	**1**	
**MS 5%; C vs H**	**Dunn's**	**<0.0001**	**0.75**		**1**
MS 5%; H vs M	Dunn's	>0.9999		1	1
**MS 10%; all groups**	**Kruskal--Wallis**	**<0.0001**	**0.75**	**1**	**1**
**MS 10%; C vs M**	**Dunn's**	**<0.0001**	**0.75**	**1**	
**MS 10%; C vs H**	**Dunn's**	**<0.0001**	**0.75**		**1**
MS 10%; H vs M	Dunn's	>0.9999		1	1
**Hexanal 5%; all groups**	**Kruskal--Wallis**	**0.0016**	**1**	**1**	**1**
Hexanal 5%; C vs M	Dunn's	0.4815	1	1	
**Hexanal 5%; C vs H**	**Dunn's**	**0.0014**	**1**		**1**
Hexanal 5%; H vs M	Dunn's	0.1031		1	1
**Hexanal 10%; all groups**	**Kruskal--Wallis**	**0.0001**	**1**	**1**	**1**
**Hexanal 10%; C vs M**	**Dunn's**	**0.0001**	**1**	**1**	
**Hexanal 10%; C vs H**	**Dunn's**	**0.0029**	**1**		**1**
Hexanal 10%; H vs M	Dunn's	0.81		1	1
**IAA 5%; all groups**	**Kruskal--Wallis**	**<0.0001**	**1**	**1**	**1**
**IAA 5%; C vs M**	**Dunn's**	**0.0026**	**1**	**1**	
**IAA 5%; C vs H**	**Dunn's**	**<0.0001**	**1**		**1**
IAA 5%; H vs M	Dunn's	0.4956		1	1
**IAA 10%; all groups**	**Kruskal--Wallis**	**0.0007**	**1**	**1**	**1**
**IAA 10%; C vs M**	**Dunn's**	**0.0007**	**1**	**1**	
**IAA 10%; C vs H**	**Dunn's**	**0.01**	**1**		**1**
IAA 10%; H vs M	Dunn's	0.8374		1	1

Comparisons of excitatory response reliability from control, mint-, and hexanal-exposed groups. Data shown in Figure 6.

### Odorant exposure changes odor tuning

**Figure 7.**
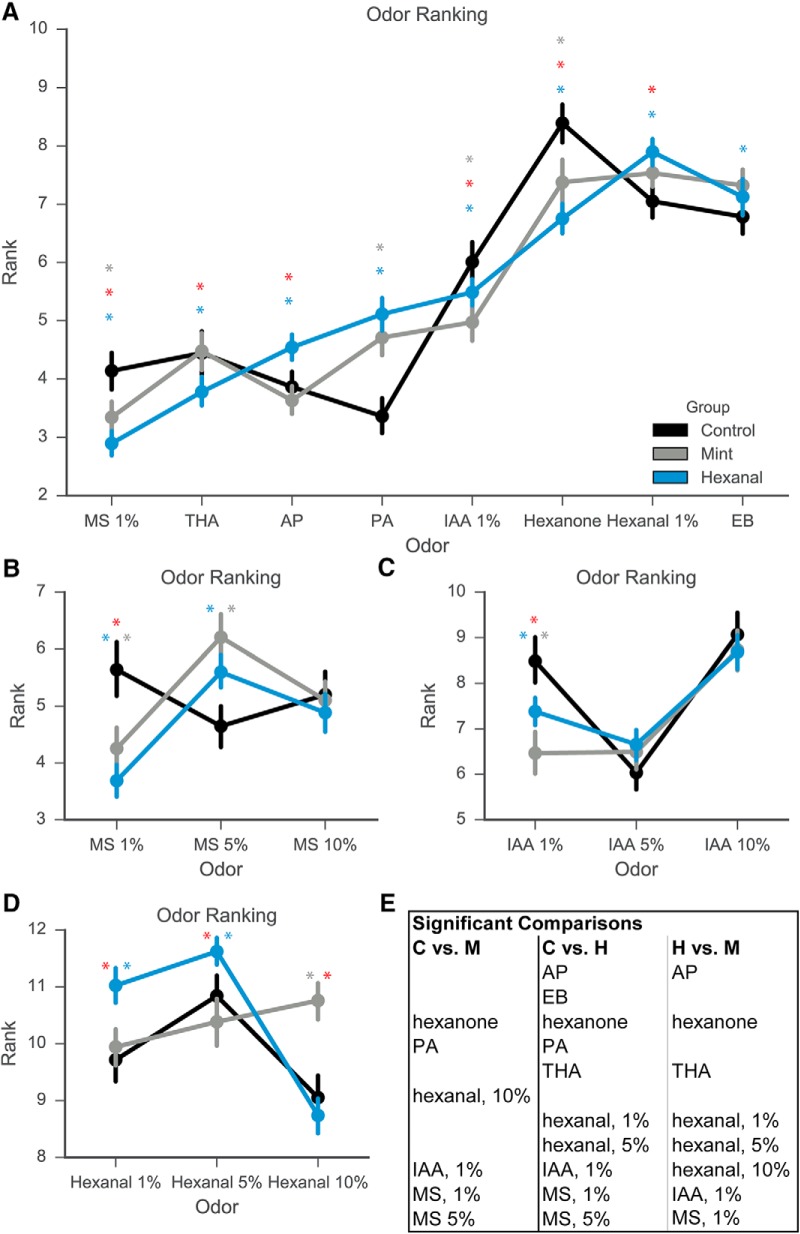
Odor ranking changes for specific odorants following odor exposure. ***A***, Response rank for each odor calculated on a cell-by-cell basis. Odorants at 1% concentration displayed. ***B***-***-D***, Response rank for multiple concentrations of MS (***B***), IAA (***C***), and hexanal (***D***). ***E***, Table of significant differences in odorant ranks for each group comparison. Gray asterisk, statistically significant difference between mint and control groups; blue asterisk, statistically significant difference between hexanal and control; red asterisk, statistically significant difference between hexanal and mint.

Given the changes in excitatory response amplitude, number, and reliability, we next investigated whether chronic early odorant exposure also changes the odor tuning curve of cells from each exposure group. Because of the high dimensionality of odorant stimuli, we used a metric of odorant response ranking to construct a tuning curve based on the stimuli in our odor panel that can then be used to compare relative responses across exposure groups. For each cell, we assigned ranks to each odorant based on the average odor-evoked response amplitude. The odorant that evoked the highest response from the cell was assigned a rank of 14, while the odorant that evoked the lowest response from the cell was assigned a rank of 1. A nonresponse to an odorant was scored as 0 amplitude and ranked accordingly, depending on if there were inhibitory and excitatory responses within the same cell. If the cell only exhibited excitatory responses, then the nonresponse to an odorant was given the rank of 1. If two or more odorants elicited the same amplitude of odor-evoked response, such as a nonresponse, those odorants were given the same ranking. There were significant differences in the rank of specific odorants between control, mint-exposed, and hexanal-exposed groups (Fig. 7). Rather than graphically denote odorants that were different, we have listed these in table form in Figure 7*E*. For clarity, results of statistical comparisons and descriptive statistics are listed in Table 5. The control group (*n* = 225 cells) differed from the mint-exposed group (*n* = 269 cells) on six out of 14 possible odorant/concentration combinations (Kruskal--Wallis test followed by Dunn’s multiple comparisons test). The control group differed from the hexanal-exposed group (*n* = 369 cells) on 10 out of 14 odorant/concentration combinations. Mint-exposed and hexanal-exposed groups differed on eight of 14 odorant/concentration combinations. These data show that food-based prenatal and postnatal odorant exposure does change MC tuning curves, as demonstrated by comparisons of individual odor ranks. There are also odor-specific differences between the mint-exposed and hexanal-exposed groups, suggesting that conditioning odorant identity could impact resultant changes in odorant response ranking.

**Table 5.** Comparison of odor ranks

Comparison	Test	*p* value	Significant	Median control (C)	Median mint (M)	Median hexanal (H)
**Response rank**				****C;******N******= 225****	****M;******N******= 268****	****H;******N******= 369****
**AP all groups**	**Kruskal--Wallis**	**<0.0001**	**Yes**	**5**	**4**	**6**
AP; C vs M	Dunn's	0.417	No	5	4	
**AP; C vs H**	**Dunn's**	**0.0002**	**Yes**	**5**		**6**
**AP; H vs M**	**Dunn's**	**<0.0001**	**Yes**		**4**	**6**
**EB all groups**	**Kruskal--Wallis**	**0.0176**	**Yes**	**10**	**10**	**11**
EB; C vs M	Dunn's	0.2086	No	10	10	
**EB; C vs H**	**Dunn's**	**0.0138**	**Yes**	**10**		**11**
EB; H vs M	Dunn's	>0.9999	No		10	11
**Hexanal 1%; all groups**	**Kruskal--Wallis**	**<0.0001**	**Yes**	**10**	**10**	**12**
Hexanal 1%; C vs M	Dunn's	>0.9999	No	10	10	
**Hexanal 1%; C vs H**	**Dunn's**	**<0.0001**	**Yes**	**10**		**12**
**Hexanal 1%; H vs M**	**Dunn's**	**<0.0001**	**Yes**		**10**	**12**
**Hexanone; all groups**	**Kruskal--Wallis**	**<0.0001**	**Yes**	**13**	**11**	**11**
**Hexanone; C vs M**	**Dunn's**	**<0.0001**	**Yes**	**13**	**11**	
**Hexanone; C vs H**	**Dunn's**	**<0.0001**	**Yes**	**13**		**11**
**Hexanone; H vs M**	**Dunn's**	**0.0001**	**Yes**		**11**	**11**
**IAA 1% all groups**	**Kruskal--Wallis**	**<0.0001**	**Yes**	**9**	**6**	**7**
**IAA 1%; C vs M**	**Dunn's**	**<0.0001**	**Yes**	**9**	**6**	
**IAA 1%; C vs H**	**Dunn's**	**0.0004**	**Yes**	**9**		**7**
**IAA 1%; H vs M**	**Dunn's**	**0.0065**	**Yes**		**6**	**7**
**MS 1% all groups**	**Kruskal--Wallis**	**<0.0001**	**Yes**	**5**	**3**	**3**
**MS 1%; C vs M**	**Dunn's**	**<0.0001**	**Yes**	**5**	**3**	
**MS 1%; C vs H**	**Dunn's**	**<0.0001**	**Yes**	**5**		**3**
**MS 1%; H vs M**	**Dunn's**	**0.0391**	**Yes**		**3**	**3**
**PA all groups**	**Kruskal--Wallis**	**<0.0001**	**Yes**	**4**	**5**	**7**
**PA; C vs M**	**Dunn's**	**<0.0001**	**Yes**	**4**	**5**	
**PA; C vs H**	**Dunn's**	**<0.0001**	**Yes**	**4**		**7**
PA; H vs M	Dunn's	0.0908	No		5	7
**THA all groups**	**Kruskal--Wallis**	**0.0028**	**Yes**	**5**	**5**	**4**
THA; C vs M	Dunn's	>0.9999	No	5	5	
**THA; C vs H**	**Dunn's**	**0.0234**	**Yes**	**5**		**4**
**THA; H vs M**	**Dunn's**	**0.0071**	**Yes**		**5**	**4**
**MS 5%; all groups**	**Kruskal--Wallis**	**<0.0001**	**Yes**	**4**	**6**	**5**
**MS 5%; C vs M**	**Dunn's**	**<0.0001**	**Yes**	**4**	**6**	
**MS 5%; C vs H**	**Dunn's**	**0.0001**	**Yes**	**4**		**5**
MS 5%; H vs M	Dunn's	0.4328	No		6	5
MS 10%; all groups	Kruskal--Wallis	0.0731	No	5	5	4
MS 10%; C vs M	Dunn's	>0.9999	No	5	5	
MS 10%; C vs H	Dunn's	0.1503	No	5		4
MS 10%; H vs M	Dunn's	0.1864	No		5	4
IAA 5%; all groups	Kruskal--Wallis	0.1561	No	7	7	6
IAA 5%; C vs M	Dunn's	0.3965	No	7	7	
IAA 5%; C vs H	Dunn's	0.1912	No	7		6
IAA 5%; H vs M	Dunn's	>0.9999	No		7	6
IAA 10%; all groups	Kruskal--Wallis	0.3245	No	10	9	9
IAA 10%; C vs M	Dunn's	0.6739	No	10	9	
IAA 10%; C vs H	Dunn's	0.4643	No	10		9
IAA 10%; H vs M	Dunn's	>0.9999	No		9	9
**Hexanal 5%; all groups**	**Kruskal--Wallis**	**<0.0001**	**Yes**	**12**	**12**	**12**
Hexanal 5%; C vs M	Dunn's	>0.9999	No	12	12	
**Hexanal 5%; C vs H**	**Dunn's**	**0.0012**	**Yes**	**12**		**12**
**Hexanal 5%; H vs M**	**Dunn's**	**<0.0001**	**Yes**		**12**	**12**
**Hexanal 10%; all groups**	**Kruskal--Wallis**	**<0.0001**	**Yes**	**9**	**12**	**9**
**Hexanal 10%; C vs M**	**Dunn's**	**<0.0001**	**Yes**	**9**	**12**	
Hexanal 10%; C vs H	Dunn's	0.7317	No	9		9
**Hexanal 10%; H vs M**	**Dunn's**	**<0.0001**	**Yes**		**12**	**9**

Statistical test results of odor ranks between control, mint-, and hexanal-exposed groups. Data shown in Figure 7.

**Table 6.** Statistical values

Statistical values	Data structure	Type of test	Power
**For all multiple comparisons tests, reported p is adjusted *p* value**			
Figure 2			
Distribution of responses; all groups; *p* = 6.65E-199	Non-normal	Kruskal--Wallis	KW statistic: 912.64
Distribution of responses; C vs M; *p* approximates 0	Non-normal	Tukey's	95% CI: -1.8211 to -1.4263
Distribution of responses; C vs H; *p* approximates 0	Non-normal	Tukey's	95% CI: -2.5607 to -2.1911
Distribution of responses; H vs M; *p* approximates 0	Non-normal	Tukey's	95% CI: -0.9273 to -0.577
Figure 3			
Excitation amplitude; IAA 10%; all groups; *p* < 0.0001	Non-normal	Kruskal--Wallis	
Excitation amplitude; IAA 10%; C (med: 0.216) vs M (0.3547); *p* < 0.0001	Non-normal	Tukey's	
Excitation amplitude; IAA 10%; C vs H (med: 0.3697); *p* < 0.0001	Non-normal	Tukey's	
Excitation amplitude; IAA 10%; H vs M; *p* = 0.0842	Non-normal	Tukey's	
Excitation amplitude; IAA 1%; all groups; *p* < 0.0001	Non-normal	Kruskal--Wallis	
Excitation amplitude; IAA 1%; C (med: 0.2018) vs M (0.2389); *p* = 0.0656	Non-normal	Tukey's	
Excitation amplitude; IAA 1%; C vs H (0.3306); *p* < 0.0001	Non-normal	Tukey's	
Excitation amplitude; IAA 1%; H vs M; *p* = 0.0001	Non-normal	Tukey's	
Excitation amplitude; AP; all groups; *p* < 0.0001	Non-normal	Kruskal--Wallis	
Excitation amplitude; AP; C (med: 0.1081) vs M (0.1837); *p* < 0.0001	Non-normal	Tukey's	
Excitation amplitude; AP; C vs H (med: 0.2822); *p* < 0.0001	Non-normal	Tukey's	
Excitation amplitude; AP; H vs M; *p* < 0.0001	Non-normal	Tukey's	
Excitation amplitude; MS 10%; all groups; *p* < 0.0001	Non-normal	Kruskal--Wallis	
Excitation amplitude; MS 10%; C (med: 0.1026) vs M (0.1961); *p* < 0.0001	Non-normal	Tukey's	
Excitation amplitude; MS 10%; C vs H (med: 0.2566); *p* < 0.0001	Non-normal	Tukey's	
Excitation amplitude; MS 10%; H vs M; *p* = 0.0022	Non-normal	Tukey's	
Excitation amplitude; IAA 5%; all groups; *p* < 0.0001	Non-normal	Kruskal--Wallis	
Excitation amplitude; IAA 5%; C (med: 0.1404) vs M (0.2386); *p* < 0.0001	Non-normal	Tukey's	
Excitation amplitude; IAA 5%; C vs H (med: 0.2973); *p* < 0.0001	Non-normal	Tukey's	
Excitation amplitude; IAA 5%; H vs M; *p* = 0.0022	Non-normal	Tukey's	
Excitation amplitude; Hexanal 1%; all groups; *p* < 0.0001	Non-normal	Kruskal--Wallis	
Excitation amplitude; Hexanal 1%; C (med: 0.2409) vs M (0.3421); *p* < 0.0001	Non-normal	Tukey's	
Excitation amplitude; Hexanal 1%; C vs H (med: 0.4718); *p* < 0.0001	Non-normal	Tukey's	
Excitation amplitude; Hexanal 1%; H vs M; *p* = 0.0001	Non-normal	Tukey's	
Excitation amplitude; EB; all groups; *p* < 0.0001	Non-normal	Kruskal--Wallis	
Excitation amplitude; EB; C (med: 0.2262) vs M (0.3254); *p* < 0.0001	Non-normal	Tukey's	
Excitation amplitude; EB; C vs H (0.3204); *p* < 0.0001	Non-normal	Tukey's	
Excitation amplitude; EB; H vs M; *p* = 0.5796	Non-normal	Tukey's	
Excitation amplitude; MS 1%; all groups; *p* < 0.0001	Non-normal	Kruskal--Wallis	
Excitation amplitude; MS 1%; C (med: 0.1167) vs M (0.1641); *p* = 0.0027	Non-normal	Tukey's	
Excitation amplitude; MS 1%; C vs H (med: 0.1933); *p* < 0.0001	Non-normal	Tukey's	
Excitation amplitude; MS 1%; H vs M; *p* = 0.0224	Non-normal	Tukey's	
Excitation amplitude; PA; all groups; *p* < 0.0001	Non-normal	Kruskal--Wallis	
Excitation amplitude; PA; C (med: 0.0733) vs M (0.2004); *p* < 0.0001	Non-normal	Tukey's	
Excitation amplitude; PA; C vs H (med: 0.2511); *p* < 0.0001	Non-normal	Tukey's	
Excitation amplitude; PA; H vs M; *p* = 0.0224	Non-normal	Tukey's	
Excitation amplitude; MS 5%; all groups; *p* < 0.0001	Non-normal	Kruskal--Wallis	
Excitation amplitude; MS 5%; C (med: 0.0777) vs M (0.1796); *p* < 0.0001	Non-normal	Tukey's	
Excitation amplitude; MS 5%; C vs H (med: 0.2503); *p* < 0.0001	Non-normal	Tukey's	
Excitation amplitude; MS 5%; H vs M; *p* = 0.6994	Non-normal	Tukey's	
Excitation amplitude; Hexanone; all groups; *p* < 0.0001	Non-normal	Kruskal--Wallis	
Excitation amplitude; Hexanone; C (med: 0.3185) vs M (0.4161); *p* < 0.0001	Non-normal	Tukey's	
Excitation amplitude; Hexanone; C vs H (med: 0.421); *p* < 0.0001	Non-normal	Tukey's	
Excitation amplitude; Hexanone; H vs M; *p* = 0.6994	Non-normal	Tukey's	
Excitation amplitude; Hexanal 10%; all groups; *p* < 0.0001	Non-normal	Kruskal--Wallis	
Excitation amplitude; Hexanal 10%; C (med: 0.2438) vs M (0.3768); *p* < 0.0001	Non-normal	Tukey's	
Excitation amplitude; Hexanal 10%; C vs H (med: 0.3778); *p* < 0.0001	Non-normal	Tukey's	
Excitation amplitude; Hexanal 10%; H vs M; *p* = 0.9842	Non-normal	Tukey's	
Excitation amplitude; THA; all groups; *p* < 0.0001	Non-normal	Kruskal--Wallis	
Excitation amplitude; THA; C (med: 0.1171) vs M (0.2183); *p* < 0.0001	Non-normal	Tukey's	
Excitation amplitude; THA; C vs H (med: 0.2415); *p* < 0.0001	Non-normal	Tukey's	
Excitation amplitude; THA; H vs M; *p* = 0.2338	Non-normal	Tukey's	
Excitation amplitude; Hexanal 5%; all groups; *p* < 0.0001	Non-normal	Kruskal--Wallis	
Excitation amplitude; Hexanal 5%; C (med: 0.2758) vs M (0.3534); *p* = 0.0001	Non-normal	Tukey's	
Excitation amplitude; Hexanal 5%; C vs H (med: 0.5212); *p* < 0.0001	Non-normal	Tukey's	
Excitation amplitude; Hexanal 5%; H vs M; *p* < 0.0001	Non-normal	Tukey's	
Figure 4**, concentration comparisons**	Non-normal		
**Control group**	Non-normal		
Excitation amplitude; MS; all conc.; *p* = 0.0586	Non-normal	Kruskal--Wallis	
Excitation amplitude; MS; 1% (med: 0.1167) vs 5% (0.0777); *p* = 0.0454	Non-normal	Tukey's	
Excitation amplitude; MS; 1% vs 10% (med: 0.1026); *p* = 0.4478	Non-normal	Tukey's	
Excitation amplitude; MS; 5% vs 10%; *p* = 0.4695	Non-normal	Tukey's	
Excitation amplitude; IAA; all conc.; *p* < 0.0001	Non-normal	Kruskal--Wallis	
Excitation amplitude; IAA; 1% (med: 0.2018) vs 5% (0.1404); *p* = 0.0027	Non-normal	Tukey's	
Excitation amplitude; IAA; 1% vs 10% (med: 0.216); *p* = 0.6607	Non-normal	Tukey's	
Excitation amplitude; IAA; 5% vs 10%; *p* = 0.0001	Non-normal	Tukey's	

Excitation amplitude; hexanal; all conc.; *p* = 0.0214	Non-normal	Kruskal--Wallis	
Excitation amplitude; hexanal; 1% (med: 0.2409) vs 5% (0.2758); *p* = 0.1412	Non-normal	Tukey's	
Excitation amplitude; hexanal; 1% vs 10% (med: 0.2438); *p* = 0.6965	Non-normal	Tukey's	
Excitation amplitude; hexanal; 5% vs 10%; *p* = 0.0189	Non-normal	Tukey's	
**Mint group**			
Excitation amplitude; MS; all conc.; *p* = 0.206	Non-normal	Kruskal--Wallis	
Excitation amplitude; MS; 1% (med: 0.1641) vs 5% (0.1796); *p* = 0.0179	Non-normal	Tukey's	
Excitation amplitude; MS; 1% vs 10% (med: 0.1961); *p* = 0.1441	Non-normal	Tukey's	
Excitation amplitude; MS; 5% vs 10%; *p* = 0.6787	Non-normal	Tukey's	
Excitation amplitude; IAA; all conc.; *p* < 0.0001	Non-normal	Kruskal--Wallis	
Excitation amplitude; IAA; 1% (med: 0.2389) vs 5% (0.2386); *p* = 0.9919	Non-normal	Tukey's	
Excitation amplitude; IAA; 1% vs 10% (med: 0.3547); *p* = 0.0001	Non-normal	Tukey's	
Excitation amplitude; IAA; 5% vs 10%; *p* = 0.0001	Non-normal	Tukey's	
Excitation amplitude; hexanal; all conc.; *p* = 0.3426	Non-normal	Kruskal--Wallis	
Excitation amplitude; hexanal; 1% (med: 0.3421) vs 5% (0.3534); *p* = 0.6541	Non-normal	Tukey's	
Excitation amplitude; hexanal; 1% vs 10% (med: 0.3768); *p* = 0.3137	Non-normal	Tukey's	
Excitation amplitude; hexanal; 5% vs 10%; *p* = 0.8335	Non-normal	Tukey's	
**Hexanal group**			
Excitation amplitude; MS; all conc.; *p* = 0.0184	Non-normal	Kruskal--Wallis	
Excitation amplitude; MS; 1% (med: 0.1933) vs 5% (0.2503); *p* = 0.0136	Non-normal	Tukey's	
Excitation amplitude; MS; 1% vs 10% (med: 0.2566); *p* = 0.2275	Non-normal	Tukey's	
Excitation amplitude; MS; 5% vs 10%; *p* = 0.4712	Non-normal	Tukey's	
Excitation amplitude; IAA; all conc.; *p* = 0.0433	Non-normal	Kruskal--Wallis	
Excitation amplitude; IAA; 1% (med: 0.3306) vs 5% (0.2973); *p* = 0.7292	Non-normal	Tukey's	
Excitation amplitude; IAA; 1% vs 10% (0.3697); *p* = 0.2089	Non-normal	Tukey's	
Excitation amplitude; IAA; 5% vs 10%; *p* = 0.0382	Non-normal	Tukey's	
Excitation amplitude; hexanal; all conc.; *p* < 0.0001	Non-normal	Kruskal--Wallis	
Excitation amplitude; hexanal; 1% (0.4718) vs 5% (0.5212); *p* = 0.2589	Non-normal	Tukey's	
Excitation amplitude; hexanal; 1% vs 10% (0.3778); *p* = 0.0127	Non-normal	Tukey's	
Excitation amplitude; hexanal; 5% vs 10%; *p* < 0.0001	Non-normal	Tukey's	
Figure 5			
Ratio of excitatory events; all odors; *p* < 0.0001	Normal	ANOVA	
Ratio of excitatory events; all odors: C (med: 0.88) vs M (0.9235); *p* < 0.0001	Normal	Tukey's	
Ratio of excitatory events; all odors: C vs H (med: 0.9377); *p* < 0.0001	Normal	Tukey's	
Ratio of excitatory events; all odors: H vs M; *p* = 0.1313	Normal	Tukey's	
Ratio of activating odors; all groups; *p* < 0.0001	Non-normal	Kruskal--Wallis	
Ratio of activating odors; C (med: 13) vs, M (14); *p* < 0.0001	Non-normal	Dunn's	
Ratio of activating odors; C vs H (med: 14); *p* < 0.0001	Non-normal	Dunn's	
Ratio of activating odors; H vs M; p>0.9999	Non-normal	Dunn's	
Figure 6			
Successes; AP; all groups; *p* < 0.0001	Non-normal	Kruskal--Wallis	
Successes; AP; C (med: 0.75) vs M (1); *p* < 0.0001	Non-normal	Dunn's	
Successes; AP; C vs H (med: 1); *p* < 0.0001	Non-normal	Dunn's	
Successes; AP; H vs M; *p* = 0.0029	Non-normal	Dunn's	
Successes; EB; all groups; *p* = 0.0181	Non-normal	Kruskal--Wallis	
Successes; EB; C (med: 1) vs M (1); *p* = 0.0151	Non-normal	Dunn's	
Successes; EB; C vs H (1); *p* = 0.5476	Non-normal	Dunn's	
Successes; EB; H vs M; *p* = 0.2351	Non-normal	Dunn's	
Successes; Hexanal 1%; all groups; *p* = 0.0596	Non-normal	Kruskal--Wallis	
Successes; Hexanal 1%; C (med: 1) vs M (med: 1); *p* = 0.7821	Non-normal	Dunn's	
Successes; Hexanal 1%; C vs H (med: 1); *p* = 0.0562	Non-normal	Dunn's	
Successes; Hexanal 1%; H vs M; *p* = 0.6464	Non-normal	Dunn's	
Successes; Hexanone; all groups; *p* = 0.1165	Non-normal	Kruskal--Wallis	
Successes; Hexanone; C (med: 1) vs M (1); *p* = 0.1277	Non-normal	Dunn's	
Successes; Hexanone; C vs H (1); *p* = 0.3668	Non-normal	Dunn's	
Successes; Hexanone; H vs M; p>0.9999	Non-normal	Dunn's	
Successes; IAA 1%; all groups; *p* = 0.0902	Non-normal	Kruskal--Wallis	
Successes; IAA 1%; C (med: 1) vs M (1); *p* = 0.4431	Non-normal	Dunn's	
Successes; IAA 1%; C vs H (1); *p* = 0.0867	Non-normal	Dunn's	
Successes; IAA 1%; H vs M; *p* > 0.9999	Non-normal	Dunn's	
Successes; MS 1%; all groups; *p* = 0.0139	Non-normal	Kruskal--Wallis	
Successes; MS 1%; C (med: 0.75) vs M (1); *p* = 0.0621	Non-normal	Dunn's	
Successes; MS 1%; C vs H (1); *p* = 0.0144	Non-normal	Dunn's	
Successes; MS 1%; H vs M; *p* > 0.9999	Non-normal	Dunn's	
Successes; PA; all groups; *p* < 0.0001	Non-normal	Kruskal--Wallis	
Successes; PA; C (med: 0.75) vs M (1); *p* < 0.0001	Non-normal	Dunn's	
Successes; PA; C vs H (1); *p* < 0.0001	Non-normal	Dunn's	
Successes; PA; H vs M; *p* = 0.0019	Non-normal	Dunn's	
Successes; THA; all groups; *p* < 0.0001	Non-normal	Kruskal--Wallis	
Successes; THA; C (med: 0.75); vs M (1); *p* < 0.0001	Non-normal	Dunn's	
Successes; THA; C vs H (1); *p* < 0.0001	Non-normal	Dunn's	
Successes; THA; H vs M; *p* = 0.9588	Non-normal	Dunn's	
Successes; MS 5%; all groups; *p* < 0.0001	Non-normal	Kruskal--Wallis	

Successes; MS 5%; C (med: 0.75) vs M (1); *p* < 0.0001	Non-normal	Dunn's	
Successes; MS 5%; C vs H (med: 1); *p* < 0.0001	Non-normal	Dunn's	
Successes; MS 5%; H vs M; *p* > 0.9999	Non-normal	Dunn's	
Successes; MS 10%; all groups; *p* < 0.0001	Non-normal	Kruskal--Wallis	
Successes; MS 10%; C (med: 0.75) vs M (1); *p* < 0.0001	Non-normal	Dunn's	
Successes; MS 10%; C vs H (med: 1); *p* < 0.0001	Non-normal	Dunn's	
Successes; MS 10%; H vs M; *p* > 0.9999	Non-normal	Dunn's	
Successes; Hexanal 5%; all groups; *p* = 0.0016	Non-normal	Kruskal--Wallis	
Successes; Hexanal 5%; C (med: 1) vs M (1); *p* = 0.4815	Non-normal	Dunn's	
Successes; Hexanal 5%; C vs H (med: 1); *p* = 0.0014	Non-normal	Dunn's	
Successes; Hexanal 5%; H vs M; *p* = 0.1031	Non-normal	Dunn's	
Successes; Hexanal 10%; all groups; *p* = 0.0001	Non-normal	Kruskal--Wallis	
Successes; Hexanal 10%; C (med: 1) vs M (1); *p* = 0.0001	Non-normal	Dunn's	
Successes; Hexanal 10%; C vs H (med: 1); *p* = 0.0029	Non-normal	Dunn's	
Successes; Hexanal 10%; H vs M; *p* = 0.81	Non-normal	Dunn's	
Successes; IAA 5%; all groups; *p* < 0.0001	Non-normal	Kruskal--Wallis	
Successes; IAA 5%; C (med: 1) vs M (1); *p* = 0.0026	Non-normal	Dunn's	
Successes; IAA 5%; C vs H (med: 1); *p* < 0.0001	Non-normal	Dunn's	
Successes; IAA 5%; H vs M; *p* = 0.4956	Non-normal	Dunn's	
Successes; IAA 10%; all groups; *p* = 0.0007	Non-normal	Kruskal--Wallis	
Successes; IAA 10%; C (med: 1) vs M (1); *p* = 0.0007	Non-normal	Dunn's	
Successes; IAA 10%; C vs H (med: 1); *p* = 0.01	Non-normal	Dunn's	
Successes; IAA 10%; H vs M; *p* = 0.8374	Non-normal	Dunn's	
Successes; All odors combined; all groups; *p* < 0.0001	Non-normal	Kruskal--Wallis	
Successes; All odors combined; C (med: 1) vs M (1); *p* < 0.0001	Non-normal	Dunn's	
Successes; All odors combined; C vs H (med: 1); *p* < 0.0001	Non-normal	Dunn's	
Successes; All odors combined; H vs M, *p* = 0.0836	Non-normal	Dunn's	
Figure 7			
Comparison of ranks; AP; all groups; *p* < 0.0001	Non-normal	Kruskal--Wallis	
Comparison of ranks; AP; C (med: 5) vs M (4); *p* = 0.4176	Non-normal	Dunn's	
Comparison of ranks; AP; C vs H (med: 6); *p* = 0.0002	Non-normal	Dunn's	
Comparison of ranks; AP; H vs M; *p* < 0.0001	Non-normal	Dunn's	
Comparison of ranks; EB; all groups; *p* = 0.0176	Non-normal	Kruskal--Wallis	
Comparison of ranks; EB; C (med: 10) vs M (10); *p* = 0.2086	Non-normal	Dunn's	
Comparison of ranks; EB; C vs H (med: 11); *p* = 0.0138	Non-normal	Dunn's	
Comparison of ranks; EB; H vs M; p>0.9999	Non-normal	Dunn's	
Comparison of ranks; Hex 1%; all groups; *p* < 0.0001	Non-normal	Kruskal--Wallis	
Comparison of ranks; Hex 1%; C (med: 10) vs M (10); p>0.9999	Non-normal	Dunn's	
Comparison of ranks; Hex 1%; C vs H (med: 12); *p* < 0.0001	Non-normal	Dunn's	
Comparison of ranks; Hex 1%; H vs M; *p* < 0.0001	Non-normal	Dunn's	
Comparison of ranks; Hexanone; all groups; *p* < 0.0001	Non-normal	Kruskal--Wallis	
Comparison of ranks; Hexanone; C (med: 13) vs M (11); *p* < 0.0001	Non-normal	Dunn's	
Comparison of ranks; Hexanone; C vs H (med: 11); *p* < 0.0001	Non-normal	Dunn's	
Comparison of ranks; Hexanone; H vs M; *p* = 0.0001	Non-normal	Dunn's	
Comparison of ranks; IAA 1%; all groups; *p* < 0.0001	Non-normal	Kruskal--Wallis	
Comparison of ranks; IAA 1%; C (med: 9) vs M (6); *p* < 0.0001	Non-normal	Dunn's	
Comparison of ranks; IAA 1%; C vs H (med: 7); *p* = 0.0004	Non-normal	Dunn's	
Comparison of ranks; IAA 1%; H vs M; *p* = 0.0065	Non-normal	Dunn's	
Comparison of ranks; MS 1%; all groups; *p* < 0.0001	Non-normal	Kruskal--Wallis	
Comparison of ranks; MS 1%; C (med: 5) vs M (3); *p* < 0.0001	Non-normal	Dunn's	
Comparison of ranks; MS 1%; C vs H (med: 3); *p* < 0.0001	Non-normal	Dunn's	
Comparison of ranks; MS 1%; H vs M; *p* = 0.0391	Non-normal	Dunn's	
Comparison of ranks; PA; all groups; *p* < 0.0001	Non-normal	Kruskal--Wallis	
Comparison of ranks; PA; C (med: 4) vs M (5); *p* < 0.0001	Non-normal	Dunn's	
Comparison of ranks; PA; C vs H (med: 7); *p* < 0.0001	Non-normal	Dunn's	
Comparison of ranks; PA; H vs M; *p* = 0.0908	Non-normal	Dunn's	
Comparison of ranks; THA; all groups; *p* = 0.0028	Non-normal	Kruskal--Wallis	
Comparison of ranks; THA; C (med: 5) vs M (5); p>0.9999	Non-normal	Dunn's	
Comparison of ranks; THA; C vs H (med: 4); *p* = 0.0234	Non-normal	Dunn's	
Comparison of ranks; THA; H vs M; *p* = 0.0071	Non-normal	Dunn's	
Comparison of ranks; MS 5%; all groups; *p* < 0.0001	Non-normal	Kruskal--Wallis	
Comparison of ranks; MS 5%; C (med: 4) vs M (6); *p* < 0.0001	Non-normal	Dunn's	
Comparison of ranks; MS 5%; C vs H (med: 5); *p* = 0.0001	Non-normal	Dunn's	
Comparison of ranks; MS 5%; H vs M; *p* = 0.4328	Non-normal	Dunn's	
Comparison of ranks; MS 10%; all groups; *p* = 0.0731	Non-normal	Kruskal--Wallis	
Comparison of ranks; MS 10%; C (med: 5) vs M (5); p>0.999	Non-normal	Dunn's	
Comparison of ranks; MS 10%; C vs H (4); *p* = 0.1503	Non-normal	Dunn's	
Comparison of ranks; MS 10%; H vs M; *p* = 0.1864	Non-normal	Dunn's	
Comparison of ranks; IAA 5%; all groups; *p* = 0.1561	Non-normal	Kruskal--Wallis	
Comparison of ranks; IAA 5%; C (med: 7) vs M (7); *p* = 0.3965	Non-normal	Dunn's	
Comparison of ranks; IAA 5%; C vs H (med: 6); *p* = 0.1912	Non-normal	Dunn's	
Comparison of ranks; IAA 5%; H vs M; p>0.9999	Non-normal	Dunn's	
Comparison of ranks; IAA 10%; all groups; *p* = 0.3245	Non-normal	Kruskal--Wallis	

Comparison of ranks; IAA 10%; C (med: 10) vs M (9); *p* = 0.6739	Non-normal	Dunn's	
Comparison of ranks; IAA 10%; C vs H (med: 9); *p* = 0.4643	Non-normal	Dunn's	
Comparison of ranks; IAA 10%; H vs M; *p* > 0.999	Non-normal	Dunn's	
Comparison of ranks; Hex 5%; all groups; *p* < 0.0001	Non-normal	Kruskal--Wallis	
Comparison of ranks; Hex 5%; C (med: 12) vs M (12); *p* > 0.9999	Non-normal	Dunn's	
Comparison of ranks; Hex 5%; C vs H (med: 12); *p* = 0.0012	Non-normal	Dunn's	
Comparison of ranks; Hex 5%; H vs M; *p* < 0.0001	Non-normal	Dunn's	
Comparison of ranks; Hex 10%; all groups; *p* < 0.0001	Non-normal	Kruskal--Wallis	
Comparison of ranks; Hex 10%; C (med: 9) vs M (12); *p* < 0.0001	Non-normal	Dunn's	
Comparison of ranks; Hex 10%; C vs H (9); *p* = 0.7317	Non-normal	Dunn's	
Comparison of ranks; Hex 10%; H vs M; *p* < 0.0001	Non-normal	Dunn's	
Figure 8			
Habituation; all groups; *p* < 0.0001	Non-normal	Friedman Test	Friedman statistic: 46.52
Habituation; Pre vs 5 min Post; *p* < 0.0001	Non-normal	Tukey's	
Habituation; Pre vs 30 min Post; *p* < 0.0001	Non-normal	Tukey's	
Habituation; 5 vs 30 min Post; *p* > 0.9999	Non-normal	Tukey's	

### MCs exhibit habituation following repeated acute odor trials

**Figure 8.**
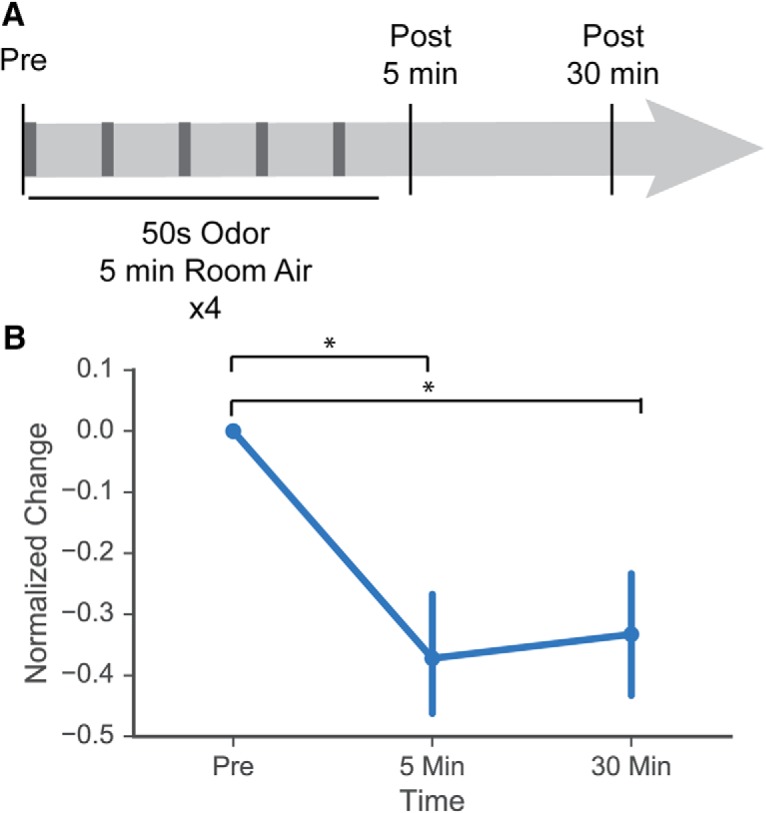
MCs display acute habituation following repeated short odor pulses. ***A***, Odor stimulus (hexanal at 1% concentration) was presented for 50 s followed by 5 min of room air, with stimulus repeated four times. MC odor-evoked response was captured before stimulus presentation, 5 min after final 50-s odor stimulus, and 30 min after final 50-s odor stimulus. ***B***, Normalized change in odor response between prestimulus and 5-min or 30-min poststimulus. Significant decrease in odor response was observed at both time points poststimulus; * indicates statistically significant comparison.

Previous work has demonstrated that repeated presentation of odors results in a decrease in amplitude of odor-evoked responses (Chaudhury et al., 2010; Ogg et al., 2015). Given that our odor exposure paradigm did not demonstrate this effect, we examined short term habituation in a subset of animals. In three animals, one from each group, we observed acute habituation of MC response. Each of these imaging sessions took place following acquisition of MC odor-evoked responses using the complete panel of odor stimuli. Using a protocol described by Chaudhury et al. (2010), we imaged hexanal-evoked activity before stimulus, presented repeated blocks of short presentations of hexanal, and imaged hexanal-evoked activity 5 and 30 min after repeated hexanal presentation (Fig. 8*A*). We found prolonged decreases in odor-evoked responses 5 min and 30 min after the stimulus presentation (*n* = 56 cells) Friedman test with Tukey’s multiple comparisons test; Median decrease in amplitude as percentage of initial “Pre” response: 37% for 5 min after stimulus and 35% for 30 min after stimulus; Pre versus 5 min: adjusted *p* < 0.0001; Pre versus 30 min: adjusted *p* < 0.0001; 5 vs 30 min: adjusted *p* > 0.9999 (Fig. 8*B*). These cells demonstrate acute habituation following repeated stimulation similar to that observed in previous studies.

## Discussion

Our data show that prenatal and early postnatal food-based odor exposure increases the amplitude, number, and reliability of excitatory MC responses *in vivo*, as measured by 2-photon calcium imaging. We observed subtle changes to MC tuning curves between exposure groups, but we did not observe any changes in MC response that were specific to the conditioned odor. The mechanisms of these widespread changes are unclear and contrast with previous work using long-term postnatal odor enrichment and acute habituation (Wilson et al., 1985, 1987; Kato et al., 2012). Key differences between our experiments and this previous work include the timing, duration, and method of odor exposure, suggesting that exposure context can modulate the effects of sensory experience on the OB. It was previously observed that prenatal and early postnatal food-based odor exposure increases the number of M/TCs associated with a glomerulus known to be activated by the conditioned odor (Liu et al., 2016). Here, we find that this exposure paradigm also induces widespread changes to odor-evoked MC responses. Such changes may be the result of several mechanisms, including the timing of odor exposure during early development, the use of food-odor association in this paradigm, and/or general sensory enrichment. Further work is necessary to determine the exact mechanisms resulting in this generalized increase in MC excitability.

### Prenatal and early postnatal odorant exposure significantly changes excitatory odor-evoked MC responses in an odor-nonspecific way

The lack of clear odor-specificity in changes following odor conditioning was surprising, given previous work showing specificity in anatomic changes following prenatal and early postnatal odorant exposure (Todrank et al., 2011; Liu et al., 2016), previous work showing that an early postnatal conditioning paradigm increases lateral inhibition (Geramita and Urban, 2016), and the observation that early postnatal odor conditioning results in a reduced response to conditioned but not other odors (Wilson et al., 1985). However, 2-DG maps of glomerular activation in rats (Glomerular Activity Response Archive, Michael Leon, gara.bio.uci.edu/index.jsp) show that both MS and hexanal activate a large number of potentially overlapping glomeruli on the dorsal OB surface, so it is possible that the widespread nature of the changes we observe in odor-evoked MC excitatory responses are due to the widespread activation of dorsal glomeruli by these exposure odorants. These changes may not be seen in areas of the OB that lack glomeruli activated by either odor. Although MS and hexanal are quite different perceptually and structurally, the lack of intuitive glomerular organization by odorant in the OB precludes us from knowing whether these two odorants activate very different sets of glomeruli. Further work is necessary to elucidate if these changes in MC response can be generalized to MCs in OB areas known to not be activated by either of these two odorants, or if odorant specific changes in excitability can be achieved with odorants known to have nonoverlapping glomerular activation maps.

Tuning curves of MC response also changed following mint and hexanal odorant exposure. Because there were also significant tuning curve differences between the mint- and hexanal-exposed groups for specific odorants in the stimulus panel, there may be relative changes in response rank specific to the identity of exposed odorant. MS and hexanal have different glomerular activation patterns, thus chronic odorant exposure may change the network of lateral inhibition and change odor-evoked response in overlapping but distinct ways. Use of a larger odor panel including ventral-activating odorants would help elucidate whether these changes in rank and in excitatory responses are connected to the identity of the odorant used for exposure.

In addition, there may be differences in the effect of early odorant exposure on male and female mice. Recent work suggests that OSN signaling differs between female and male mice – specifically, OSNs from female mice respond faster to odor presentation and are more broadly tuned than male OSNs (Kass et al., 2017). However, previous work by Liu et al. (2016) showed that early odorant exposure using the same paradigm as in this study changes the number of M/TCs connected to a single activated glomerulus, but these changes are not significantly different between male and female mice. In our data, we find that the distribution of odor-evoked excitatory MC responses is different between male hexanal-exposed and control mice but not between male mint-exposed and control mice. Contrastingly, there were significant differences in response distribution between female odor-exposed (both hexanal- and mint-exposed) and control mice. However, we only obtained data from one female control and two female odor-exposed animals from each group, insufficient subject number to draw strong conclusions about whether these changes in response distribution are due to interanimal variability or sexual dimorphism.

### Activation pattern similarity of odorant stimuli

In this study, most cells had observed above-threshold responses to most odorants in the panel (Fig. 5*E*). This difference from the sparser MC response to odorants observed previously (Kato et al., 2012; Blauvelt et al., 2013; Wachowiak et al., 2013; Roland et al., 2016) could be due to (1) the high sensitivity of GCaMP6s calcium indicator, (2) the choice of odorants within the stimulus panel, (3) the use of higher odorant concentrations in our study, and (4) the use of an anesthetized preparation. In *in vivo* studies of visual cortical neurons, use of GCaMP6s resulted in a fivefold higher rate of detection of responding neurons than GCaMP3, suggesting that the larger proportion of observed odor-evoked responses in our data set could be partially explained by use of a more sensitive calcium indicator (Chen et al., 2013). The choice of odorants used for chronic exposure and in the odor panel was deliberately focused on dorsal OB activating odorants to visualize odor-evoked responses in the cohort of cells imaged, those on the dorsal OB surface. Thus, the composition of the stimulus panel may explain why a large proportion of cells on the dorsal OB surface showed significant odor-evoked responses to many dorsally-activating odorants in the panel.

MC activity differs significantly between the awake and the anesthetized states. Kato et al. (2012), found that the MCs of anesthetized animals are more responsive, each odorant elicits response from a larger number of MCs, and the amplitude of odor-evoked responses is also greater. They also found that experience-dependent habituation of odor-evoked MC response is dependent on odor presentation in the awake state, as brief odor presentation while under anesthesia did not result in MC response habituation. Chaudhury et al. (2010), observed that acute odorant exposure causes habituation of MC response in anesthetized animals, a finding that we replicate in Figure 8. In contrast to Kato et al. (2012), our odorant exposure paradigm consists of food-based odorant exposure throughout gestation and nursing. This paradigm has been shown to induce significant changes in both glomerulus volume and the number of M/TCs connected to single glomeruli (Todrank et al., 2011; Liu et al., 2016). Todrank et al. (2011), additionally observed that odorant exposure limited to either the gestation period or the postnatal nursing period induced an increase in glomerular volume. In this study, we do not address whether the experience-dependent changes in MC response can be observed following odorant exposure limited to a specific developmental period or whether these changes can be observed in the awake animal. However, given the significant anatomic changes in the OB observed after prenatal and early postnatal odorant exposure, we predict that we would also observe an increase in excitatory odor-evoked MC response in awake mice and that this change remains robust following exposure limited to either gestation or nursing.

### Prenatal and postnatal timing of odorant exposure

The timing of our odorant exposure paradigm may be one explanation for why we find an increase rather than a decrease in excitability following exposure. Here, we use a conditioning paradigm of constant odor exposure during both gestation and the postnatal period. Todrank et al. (2011) show that exposure during either gestation or early nursing is sufficient to significantly increase the size of activated glomeruli, while Liu et al. (2016) demonstrate that this paradigm also increases the number of M/TCs connected to a single activated glomerulus. Prenatal food-based odor exposure can produce large anatomic changes in the OB circuitry. We show that this paradigm also promotes nonspecific changes to excitatory MC odor-evoked responses; these findings contrast with previous studies of odor-evoked MC responses following both acute and chronic odor exposure. However, the majority of studies analyzing experience-dependent changes to the structure and function of the OB rely on postnatal manipulations (Benson et al., 1984; Laing, 1985; Panhuber and Laing, 1987; Saghatelyan et al., 2005; Kerr and Belluscio, 2006; Woo et al., 2006; Cavallin et al., 2010; Johnson et al., 2013; Morrison et al., 2015; Geramita and Urban, 2016). Starting exposure during gestation may trigger developmental changes distinct from those observed with postnatal odor experience.

### Sensory enrichment

Generalized early postnatal sensory enrichment may be another explanation for the observed nonspecific enhanced excitatory responses. Odor deprivation during development causes significant changes to OB structure and activity. OB size, OSN activity, MC connectivity, and granule cell integration are all impacted by early nares occlusion (Benson et al., 1984; Saghatelyan et al., 2005; Cavallin et al., 2010). With regards to the anatomic effects of early postnatal chronic odor enrichment or stimulation, the consensus is less clear. A number of studies report a significant decrease in the size and density of MCs following chronic passive odor exposure (Laing, 1985; Panhuber and Laing, 1987; Woo et al., 2006; Johnson et al., 2013). However, Rosselli-Austin and Williams (1990), used scented objects in a normal cage setting to deliver chronic odor stimulation and found that mitral and granule cell numbers actually increased following neonatal odorant exposure. In addition, numbers of adult born granule cells (Rochefort et al., 2002) and dopaminergic cells (Bonzano et al., 2014) also increase following chronic postnatal odorant exposure. These results suggest that richness of the sensory environment during the neonatal period can significantly modify OB structure. Our work points to a general increase in MC excitability, as measured by the number and amplitude of excitatory odor-evoked responses. This generalized change could be due to sensory enrichment through odorant exposure during a critical period of OB development.

Enrichment can also modify the excitability of other sensory systems. Studies in cat visual cortex showed that rearing animals in an enriched environment increased the number of orientation selective cells, sharpened orientation tuning of cells, and increased responsivity of cells to light stimuli (Beaulieu and Cynader, 1990a,b). Such changes are observed during adulthood as well, following visual enrichment, adult rats with amblyopia demonstrated improved visual acuity and reduced inhibition/excitation balance in V1 (Baroncelli et al., 2012). Other sensory systems also demonstrate this feature, with sensory enrichment increasing excitatory responses and refinement of stimulus selectivity within the auditory and somatosensory systems (Coq and Xerri, 1998; Bourgeon et al., 2004; Engineer et al., 2004; Alwis and Rajan, 2013) This phenomenon also may be taking place in the OB, as we observe a generalized, nonstimulus specific increase in MC excitatory response amplitude and number following odorant exposure.

### Food-associated exposure paradigm

Lastly, food-association may contribute to the generalized increase in MC excitation. Here, we pair food with odorant. For the moth *Manduca sexta*, pairing repeated odor exposure with food resulted in an increase in responsive neurons while repeated odor exposure without food caused a decrease in responsive neurons (Daly et al., 2004). While these experiments were conducted in a different organism and used acute trials of odor pairing rather than the chronic odor exposure that we use, the *Manduca* study demonstrates that odorant context plays a role in experience-dependent modifications in response. When exposed to odorized food, mice demonstrate a distinct preference for food scented with the familiar odorant (Todrank et al., 2011; Liu et al., 2016), indicating a positive odorant association following food-based exposure. However, Wilson et al. (1985), found that pairing odor puffs with brushing, an association with a positive or attractive context, results in a decrease of excitatory responses to presentations of the exposure odorant alone. The contrasting results from studies that use different odor exposure paradigms, reward-paired or passive, suggest that context of odor presentation could play an important role in determining how the circuit remodels anatomically and functionally following repeated or chronic odor exposure.

## Conclusion

Here, we use a food-based paradigm to investigate the effects of prenatal and early postnatal odor exposure on odor-evoked responses of MCs. We find that odorant exposure heightened dorsal OB MC activity, increasing the amplitude, reliability, and the proportion of excitatory odor-evoked MC responses. We also find that odorant exposure changed the tuning curves of MCs in exposed animals. These effects were not specific to odor-evoked responses to either MS or hexanal, the odorants used for exposure. Rather, prenatal and early postnatal odorant exposure using either odorant resulted in generalized changes to MC responses across the dorsal OB and in response to all odorants in the stimulus panel. Control, hexanal-, and MS-exposed animals all demonstrated a similar decrease in odor-evoked MC responses following acute odorant habituation with hexanal, indicating that our paradigm of constant early odorant exposure does not fundamentally change mechanisms of habituation.

## Synthesis

Reviewing Editor: Anna Menini, SISSA (International School of Advances Studies) Decisions are customarily a result of the Reviewing Editor and the peer reviewers coming together and discussing their recommendations until a consensus is reached. When revisions are invited, a fact-based synthesis statement explaining their decision and outlining what is needed to prepare a revision will be listed below. The following reviewer(s) agreed to reveal their identity: Diego Restrepo, Graeme Lowe

This manuscript contributes to the understanding of plasticity in the olfactory system, showing that odor environment and experience have clear effects on physiological odor responses of neurons in the main olfactory bulb. Both reviewers have recommendations about improving the presentation of Methods, Results and Discussion. Please carefully address the specific concerns listed below.

Specific comments of reviewer 1

This manuscript makes a contribution to the understanding of plasticity in the olfactory system. The authors record odorant-induced activity in mitral cells of mice that have been exposed to odorants added to the food of the mother in the pre-natal and postnatal periods. They find an increase in mitral cell responsiveness to a subset of odorants known to elicit dorsal glomerular responses.

1. The presentation of the data would benefit by changes in the order of figures and addition of more examples. I suggest placing Figs. 4 and 5 immediately after Fig. 2 because they show the changes in DF/F elicited by the pre/post-natal exposure. I recommend to move Fig. 3 (odor ranking) to the end.

2. The procedure used for ranking in Fig. 3 was not explained in the methods. How was the ranking performed when two of the odorants did not elicit a response? In addition, this figure would benefit by displaying an example of raw traces to show how ranking was performed.

3. Given the p values shown in the tables this will likely not make a large difference. However, the authors should perform a correction for multiple comparisons.

4. The discussion needs tightening. In particular, the reference from Kato et al 2012, and references cited within that manuscript describe studies of changes in responsiveness elicited by odorant exposure. Those experiments were performed with a very different odor exposure paradigm, but it is important to discuss those data. In addition, Kato et al show a large difference between mitral cell odorant responses in awake vs. anesthetized animals, and other groups have characterized differences in M/T cell responsiveness with extracellular recordings. Would recordings in the awake mouse show a generalized increase of responsiveness of the mitral cells?

Minor

1. Line 214 “The mechanisms of these widespread changes are unclear but are surprising given previous work using postnatal odor enrichment (Wilson et al., 1985, 1987).” I do not find this surprising given the difference in context (odor in the food of the mother vs. odor exposure). Also, please reference other studies (see Kato et al 2012).

2. Line 140. This reference is not in the list: “the largest numerical rank (14)”.

3. Fig. 1 should be presented in the results.

4. Line 147. “These data show that food based prenatal and postnatal odorant exposure does change the relative odor preferences for specific odors.” This experiment does not determine odor preference.

Specific comments of reviewer 2

Prenatal and early postnatal pairing of food with odors was recently shown to induce long lasting changes in neuroanatomy of the mouse olfactory system. Food-odor exposure increased sizes of olfactory bulb glomeruli whose receptors recognized the conditioned odors (Todrank et al 2011), and increased numbers of mitral cells (MC) and tufted cells (TC) relaying output signals from these glomeruli (Liu et al 2016). In this study, the authors investigated physiological correlates of such changes by comparing odor-evoked MC activity (assayed by Ca-imaging) of control and odor-conditioned mice. Conditioning by 2 different odors was associated with alterations in MC population tuning to a panel of 8 test odors, with increased selectivity for conditioned odors. Measures of response sensitivity, reliability, and fraction of excitatory response increased overall for the panel, not only for the 2 conditioned odors. This is an unexpected and potentially novel result because previous studies predicted changes specific to glomerular pathways activated by conditioned odors.

The experimental work is well executed, technically impressive and acquired a large dataset. The reported effects are strong, clearly visualized in charts, and supported by exhaustive statistical analyses. Two limitations of the study reduce its general impact.

(1) the only treatment was continued food-odor exposure prenatal and postnatal, so it is not possible to know if the physiological effects were induced by prenatal vs. postnatal mechanisms, which could be fundamentally different. The prior study of Todrank et al 2011 separated groups with pre- vs. postnatal exposure, showing that both had effects.

(2) Interpretation of the results is limited by not knowing the glomerular affiliations of recorded MCs (viral transduction of the GCaMP reporter was non-specific). Glomerular-resolution odor maps on the dorsal olfactory bulb for the 8 odors in the panel, including the 2 conditioned ones, were not determined, so it is not possible to understand if the 'non-specific' increases in MC population responses are due to overlap of glomerular maps. This could help to interpret data in terms of previous studies showing glomerulus-specific effects.

Main comments

116-118, Fig. 2: analysis of influence of odor exposure on MC odorant responses focused on peak amplitude and integrated area-under-curve of Ca responses, which would correspond to MC action potential firing rates (= rate coding). However, olfactory coding is also thought to depend on temporal features of responses. Were kinetic or dynamic characteristics e.g. latency, rise, decay, or shapes of responses affected?

85: “Both male and female mice were used for imaging”: there are large differences in glomerular odor map responses of female vs. male mice, with broader tuning in females, c.f. Kass et al 2017. The dataset in this study is from: “5 control, 6 mint-exposed, and 6 hexanal-exposed” mice. The relatively small group sizes raise concerns about how they were partitioned by sex, and whether this led to any bias in the data analysis. Was the analysis performed to remove potential bias due to sexual dimorphism?

Minor comments.

72, 78: temperature units (C or F) should be specified

98: “Two-photon imaging was done in the MC layer”: some glomerulus-projecting deep short axon cells (GL-dSACs) reside in the underlying IPL very close or adjacent to the MCL. Can these be excluded from the cells imaged, or maybe relabeled as “putative MCs”?

102: “The dataset consists of 862 cells...”: It is not clear here - does dataset include all GCaMP6 fluorescent cells detected, or only those cells that responded to odors in at least one trial (and thus excludes completely unresponsive cells)? What fraction of all GCaMP6 fluorescent cells detected by imaging were responsive? Was the fraction increased by odor exposure (as might be predicted from Liu et al 2016)? Maybe this is already included in the analysis of proportion of successful trials (Fig. 7) but it is not clear.

107: “Traces were detrended by subtracting a polynomial fit”: Methods description needs to be more precise. What points in the traces were fitted? What order of polynomial? etc

114: “MC odor-evoked responses were dominated by excitatory responses..”: Traces in Fig. 1D show only excitatory responses (part of the 89%). So were the rest of the responses (11%) suppressive/ inhibitory? What did the traces of inhibitory responses look like?

292: “A similar mechanism may explain our findings”: It is certainly plausible that the phenomenon of food-odor pairing associative olfactory plasticity occurs across animal phyla because of its adaptive advantage. However, underlying mechanisms depend on anatomy, neurotransmitter, or neuromodulator pathways which are quite different in insects and mammals. Moreover, food-odor association in Manduca was induced in adults, not during early developmental critical period. Oddly, the author's premise of similar mechanism in adult Manduca seems to contradict their point in a previous paragraph (269-270) that “exposure during gestation may trigger developmental changes distinct from those observed with postnatal odor experience”. The previous study (Todrank et al 2011) found no effect of (food + odor) on glomerular volume in the older cohort of late-nursing mice. And obviously, any in-utero developmental mechanisms in placental mammals are unlikely to occur the non-viviparous Manduca.

Figs. 1-8: adding tick marks to the plots would provide clearer, more precise data presentations

Figs. 2A-C: adding axes and tick marks to the plots would provide clearer, more precise data presentations; presumably some of the points with DF/F < 0 are the inhibitory responses? But the position of the plots origins are not clearly defined because only tick labels are shown.

Fig. 8A: the graphically indicated time blocks are confusing, do not look to be in stated 50s/5 min ratios

Fig. 8B: is this from data pooled for all 3 animals representing different groups (control, Hexanal, Methyl salicylate food-exposure)? Was there no difference in hexanal habituation for the hexanal exposure group?
